# Supplementary material for: AF-React study: atrial fibrillation management strategies in clinical practice—retrospective longitudinal study from real-world data in Northern Portugal
Source: BMJ Open. 2021 Mar 29;11(3):e040404. doi: 10.1136/bmjopen-2020-040404 (PMC8009225; doi:10.1136/bmjopen-2020-040404)
Supplement: Author's manuscript [file bmjopen-2020-040404.draft_revisions.pdf]

BMJ Open

BMJ Open is committed to open peer review. As part of this commitment we make the peer review history of every article we publish publicly available.

When an article is published we post the peer reviewers' comments and the authors' responses online. We also post the versions of the paper that were used during peer review. These are the versions that the peer review comments apply to.

The versions of the paper that follow are the versions that were submitted during the peer review process. They are not the versions of record or the final published versions. They should not be cited or distributed as the published version of this manuscript.

BMJ Open is an open access journal and the full, final, typeset and author-corrected version of record of the manuscript is available on our site with no access controls, subscription charges or pay-per-view fees (http://bmjopen.bmj.com).

If you have any questions on BMJ Open's open peer review process please email info.bmjopen@bmj.com

BMJ Open

The AF-React study: Atrial fibrillation management strategies in clinical practice in Northern Portugal, insights from real-world data

Journal:	*BMJ Open*
Manuscript ID	bmjopen-2020-040404
Article Type:	Original research
Date Submitted by the Author:	12-May-2020
Complete List of Authors:	Silva Pinto, Susana; University of Porto Faculty of Medicine, Family Medicine Unit, Department of Community Medicine, Information and Health Decision Sciences (MEDCIDS); Center for Health Technology and Services Research<br>Teixeira, Andreia; University of Porto Faculty of Medicine, Department of Community Medicine, Information and Health Decision Sciences (MEDCIDS); Center for Health Technology and Services Research<br>Henriques, Teresa; University of Porto Faculty of Medicine, Department of Community Medicine, Information and Health Decision Sciences (MEDCIDS); Center for Health Technology and Services Research<br>Monteiro, Hugo; Research and Planning Department, Regional Health Administration of Northern, Min. of Health Portugal<br>Martins, Carlos; University of Porto Faculty of Medicine, Family Medicine Unit, Department of Community Medicine, Information and Health Decision Sciences (MEDCIDS); Center for Health Technology and Services Research
Keywords:	PRIMARY CARE, STROKE MEDICINE, Adult cardiology < CARDIOLOGY

SCHOLARONE™
Manuscripts

I, the Submitting Author has the right to grant and does grant on behalf of all authors of the Work (as defined in the below author licence), an exclusive licence and/or a non-exclusive licence for contributions from authors who are: i) UK Crown employees; ii) where BMJ has agreed a CC-BY licence shall apply, and/or iii) in accordance with the terms applicable for US Federal Government officers or employees acting as part of their official duties; on a worldwide, perpetual, irrevocable, royalty-free basis to BMJ Publishing Group Ltd ("BMJ") its licensees and where the relevant Journal is co-owned by BMJ to the co-owners of the Journal, to publish the Work in this journal and any other BMJ products and to exploit all rights, as set out in our licence.

The Submitting Author accepts and understands that any supply made under these terms is made by BMJ to the Submitting Author unless you are acting as an employee on behalf of your employer or a postgraduate student of an affiliated institution which is paying any applicable article publishing charge ("APC") for Open Access articles. Where the Submitting Author wishes to make the Work available on an Open Access basis (and intends to pay the relevant APC), the terms of reuse of such Open Access shall be governed by a Creative Commons licence – details of these licences and which Creative Commons licence will apply to this Work are set out in our licence referred to above.

Other than as permitted in any relevant BMJ Author's Self Archiving Policies, I confirm this Work has not been accepted for publication elsewhere, is not being considered for publication elsewhere and does not duplicate material already published. I confirm all authors consent to publication of this Work and authorise the granting of this licence.

For peer review only

TITLE PAGE

The AF-React study: Atrial fibrillation management strategies in clinical practice in Northern Portugal, insights from real-world data.

Pinto, Susana Silva;[1, 2,3] Email: susyapinto@gmail.com
[1]Ponte Velha Family Health Unit (ACeS Santo Tirso/Trofa), Portugal. Email:
[2]Department of Community Medicine, Information and Health Decision Sciences (MEDCIDS), Faculty of Medicine, University of Porto, Porto, Portugal.
[3]Centre for Health Technology and Services Research (CINTESIS), University of Porto, Porto, Portugal.

Teixeira, Andreia;[2, 3, 4] Email: andreiasofiat@hotmail.com
[2]Department of Community Medicine, Information and Health Decision Sciences (MEDCIDS), Faculty of Medicine, University of Porto, Porto, Portugal.
[3]Centre for Health Technology and Services Research (CINTESIS), University of Porto, Porto, Portugal.
[4]ARC4DigiT, Polytechnic Institute of Viana do Castelo, Portugal.

Henriques, Teresa S.;[2, 3] Email: teresasarhen@gmail.com
[2]Department of Community Medicine, Information and Health Decision Sciences (MEDCIDS), Faculty of Medicine, University of Porto, Porto, Portugal.
[3]Centre for Health Technology and Services Research (CINTESIS), University of Porto, Porto, Portugal.

Monteiro, Hugo;[5] Email: hfmonteiro@arsnorte.min-saude.pt
[5]Research and Planning Department, Regional Health Administration of Northern, Min. of Health Portugal.

Martins, Carlos.[2, 3] Email: carlosmartins20@gmail.com
[2]Department of Community Medicine, Information and Health Decision Sciences (MEDCIDS), Faculty of Medicine, University of Porto, Porto, Portugal.
[3]Centre for Health Technology and Services Research (CINTESIS), University of Porto, Porto, Portugal.

Corresponding Author
Susana Silva Pinto
susyapinto@gmail.com

For peer review only

ABSTRACT

Objectives

To determine the prevalence of AF and to assess how these patients are being cared for: what anticoagulants are being prescribed and are they being prescribed as recommended?

Design

Retrospective longitudinal study.

Setting

This study was conducted in Regional Health Administration of Northern Portugal.

Participants

This study using a database that included 63,526 patients with code K78 of the International Classification of Primary Care between January 2016 and December 2018.

Results

The prevalence of AF among adults over 40 years in the northern region of Portugal was 2.3% in 2016, 2.8% in 2017, and 3% in 2018. From a total of 63,526 patients, 95.8% had an indication to receive anticoagulation therapy. Of these, 44,326 (69.8%) are being treated with anticoagulants: 17,936 (40.5%) were prescribed with vitamin K antagonists and 26,390 (59.5%) with non-vitamin K antagonist anticoagulants.

Conclusions

The prevalence of AF is 3%. Here we report evidence of undertreatment, but also of overtreatment. Albeit having an indication, there is a considerable proportion of patients (26.0%) that are not anticoagulated, and among AF patients without indication to receive anticoagulation therapy, there is a considerable proportion (40.9%) receiving anticoagulants.

Keywords

atrial fibrillation, anticoagulants, prevention, stroke; primary health care.

Strengths and limitations of this study

- The main limitation of this retrospective study is that the AF assessment is based on data coded in the clinical process in Primary Health Care.
- There might be more patients in the northern region of Portugal with AF, but they are not coded at Primary Health Care and, therefore, were not included in this study.
- The lack of ICPC-2 coding for problems such as mechanical prosthesis valve and rheumatic moderate or severe mitral stenosis might be another limitation.
- There might be a small percentage of patients well medicated with vitamin K antagonists because they are in one of those situations.
- Few of the 63,526 patients will be diagnosed with atrial flutter, however for patients with atrial flutter, anticoagulant therapy is recommended according to the same risk profile used for AF.

For peer review only

For peer review only - http://bmjopen.bmj.com/site/about/guidelines.xhtml

BACKGROUND

Atrial fibrillation (AF) is the most common type of sustained cardiac arrhythmia.(1) The clinical relevance of AF is associated to the loss of effective atrial contractility resulting in deficient emptying of the left atrial appendage. This process increases the risk of thrombus formation and thromboembolic events.(1)

Due to the ageing of the population, the number of people affected by AF worldwide is projected to exceed 12 million by 2050.(2) In Portugal in 2010, the FAMA study(3) verified an overall prevalence of AF of 2.5% in a population sample older than 40 years, with a significant increase after the age of 70 years.(3)

In Portugal, despite a considerable reduction in ischemic stroke mortality below 70 years (39% reduction) in 2015, stroke remains the main cause of mortality related to vascular diseases.(4) Patients with AF have a five-fold increased risk of stroke.(5) This arrhythmia is currently responsible for about 15% of stroke cases.(3) Stroke is a major complication associated with AF, which contributes to the morbidity and mortality associated with the disease.(6)

Previous studies have reported that oral anticoagulation with vitamin K antagonists (VKA), as warfarin, reduces stroke and mortality in AF patients.(7) The non-vitamin K antagonist oral anticoagulants (NOAC) – dabigatran, rivaroxaban, apixaban, edoxaban – have been shown to be superior to warfarin in the prevention of thromboembolic events in patients with non-valvular AF, providing increased safety and an overall reduction in the number of cases of bleeding events. The cost and morbidity associated with stroke and intracranial haemorrhage are high, so the lower prevalence of these events with NOACs is one factor that might help this drug to become cost-effective relative to warfarin.(8) Furthermore, according to the Portuguese Directorate-General of Health, the reduction in ischemic stroke mortality below 70 years was due to a set of factors that highlight the contribution of the introduction into clinical practice of NOAC as an antithrombotic therapy for AF. (4)

In a cross-sectional study of patients with AF done in eight Family Health Units of Northern Portugal (the FATA study),(9) only 56.8% of patients with AF were prescribed adequate oral anticoagulation according to the recommendations of the European Society of Cardiology (ESC) Guidelines in 2010. In the SAFIRA study,(10) 56.3% of patients with AF were not anticoagulated.

There are already considerable randomised controlled trials with the NOACs. For example, the RE-LY,(11) ROCKET-AF,(12) ARISTOLETE,(13) and ENGAGE AF-TIMI 48(14) studies included 18,113, 14,264, 18,201 and 21,105 patients with AF respectively. In fact, these trials were crucial for the efficacy and safety evaluation of these drugs and their approval. However, the real-world studies might contribute with additional relevant evidence because they include epidemiological data and drug prescribing patterns for a larger number of patients in the context of daily clinical practice. In Portugal and throughout Europe, there are no real-world studies with a large database of oral anticoagulation in patients with AF.

The AF-React study aims to determine the prevalence of AF in the northern region of Portugal and to assess how patients with AF are being cared for: what anticoagulants are being prescribed and are they being prescribed as recommended?

For peer review only - http://bmjopen.bmj.com/site/about/guidelines.xhtml

METHOD

This retrospective longitudinal study was conducted using a database from the Regional Health Administration of Northern Portugal. This database included 63,526 patients with AF: all the adults (age ≥ 18 years) with the code for AF, K78, of the International Classification of Primary Care (ICPC-2)(15) as an active problem between January 2016 and December 2018.

The cardiovascular risk was defined in four stages based on SCORE (Systematic Coronary Risk Evaluation), as low, moderate, high, and very high, if less than 1%, between 1% and 5%, between 5% and 10%, and greater than or equal to 10%, respectively. The high stage was also considered both in patients with diabetes and no other cardiovascular risk factor or target organ lesion or in patients with moderate chronic kidney disease, CKD.(16,17) The very high stage was also considered in patients with documented atherosclerotic cardiovascular disease, patients with diabetes and another cardiovascular risk factor, or target organ lesion or in patients with severe CKD, or any combination of these.(16,17) The stages of CKD were defined based on the glomerular filtration rate (GFR): stage 1, GFR ≥ 90 ml/min; stage 2, GFR]90-60] ml/min; stage 3, GFR]60-30] ml/min, stage 4, GFR [30-15] ml/min; and stage 5, GFR < 15ml/min. The CHA_2DS_2-VASc score was calculated according to the ESC guidelines in 2016 for AF.(18) Oral anticoagulation was considered in a male with a CHA_2DS_2-VASc score ≥ 1 point and in a female with a score ≥ 2 points. The variables related to health problems were based on ICPC-2.(15)

The categorical variables were described using absolute and relative frequencies, n (%). The normally distributed continuous variables were described by the mean and respective standard deviation, $\bar{x} \pm s$, and by the minimum (min) and maximum (max) values. In the case of continuous variables not normally distributed, the data are presented by the median and respective interquartile range, Med [Q_1; Q_3], where Q_1 is the first quartile and Q_3 is the third quartile. The normality was verified by observing the histograms.

The database was exported to Microsoft Excel 2016®, and the statistical analysis was performed using SPSS Statistics 25.0® and software R. Data were obtained with specific filters, without aggregation at the individual level for general characterisation analysis.

The data processing was carried out through the Department of Studies and Planning of the Regional Health Administration of Northern Portugal (Ministry of Health, Portugal). The data was extracted from the server through an anonymised data processing and editing platform and delivered securely (and in accordance with legal regulations and due approval) to the principal investigator. Patients and public were not involved in the development of the research question.

For peer review only - http://bmjopen.bmj.com/site/about/guidelines.xhtml

RESULTS

A total of 63,526 patients were identified to have the diagnosis of atrial fibrillation/flutter (ICPC-2, K78 code) in the northern region of Portugal. The average age was 76 ± 10.6 years (range, to 107 years) and 53% were female. Table 1 provides a detailed characterisation of the study population. Considering a total of adults over 40 years included in the Regional Health Administration of Northern Portugal, the prevalence of AF among the adults over 40 years in the northern region of Portugal was 2.3% in 2016, 2.8% in 2017, and 3% in 2018 (Table 2).

From a total of 63,526 patients, 60,838 (95.8%) had an indication to receive anticoagulation therapy: male with CHA_2DS_2-VASc score ≥ 1 and female with CHA_2DS_2-VASc score ≥ 2. Among these 60,838 patients, 16,512 (26%) were not being treated with anticoagulants, and 79% of those 16,512 have a CHA_2DS_2-VASc score of 3 or higher.

Among the 44,326 (69.8%) being treated with anticoagulants, 17,936 (40.5%) were prescribed with VKAs and 26,390 (59.5%) with NOACs. The most commonly prescribed drug in medicated patients is warfarin (31%), followed by rivaroxaban (23.6%), apixaban (20.3%), dabigatran (12.6%), acenocoumarol (9.6%), and edoxaban (2.9%).

From the total of 30,089 males, 1658 (5.5%) had a CHA_2DS_2-VASc score of zero, and from a total of 33,437 females, 1030 (3.1%) had a CHA_2DS_2-VASc score of 1. This means that these 2688 (4.2%) patients had no indication to receive anticoagulation therapy. Of these 2688 patients, 1100 were receiving anticoagulants (Table 3).

The distributions of the different drugs by CHA_2DS_2-VASc score and gender are shown in Table 4. For males, rivaroxaban (37.1%) and apixaban (20.1%) are more often prescribed than warfarin (19%) in the group with a CHA_2DS_2-VASc score of zero. In particular, apixaban is more prescribed than rivaroxaban in groups with CHA_2DS_2-VASc scores of 5 or 6 (20.3% *vs* 19.7% and 18.4% *vs* 18%, respectively). On the other hand, acenocoumarol is more prescribed than dabigatran in the groups with CHA_2DS_2-VASc scores of 7 or 8 (12.5% *vs* 11.9% and 15.1% *vs* 3.8%, respectively). For females, apixaban is more prescribed than rivaroxaban in the groups with CHA_2DS_2-VASc scores of 7, 8, or 9 (24.9% *vs* 18.5%, 26.8% *vs* 18.8%, and 23.7% *vs* 18.6%, respectively). Furthermore, the acenocoumarol is more prescribed than dabigatran in the group a CHA_2DS_2-VASc score of 8 (10.4% *vs* 9.5%).

From 63,526 patients with AF, 17,936 (28.2%) were always prescribed with VKA, 21,854 (34.4%) were always prescribed with NOAC, 3508 (5.5%) were prescribed VKA who switched to NOAC, and 1504 (2.4%) were prescribed with NOAC who switched to another NOAC.

The prevalence of each of the pathologies studied in patients with AF is represented in Table 5. We found that the most prevalent pathologies associated with AF are hypertension (77.2%), dyslipidemia (52.1%), diabetes (28.5%), and heart failure (27.6%).

For peer review only - http://bmjopen.bmj.com/site/about/guidelines.xhtml

DISCUSSION

Summary

In the AF-React study, a prevalence of AF was 3% in 2018. A considerable proportion of patients, with indication for anticoagulation therapy, are not anticoagulated (26.0%), and among AF patients without indication to receive anticoagulation therapy, there is a considerable proportion (40.9%) receiving anticoagulants.

Comparison with existing literature

The prevalence of AF found in this study is similar to that of the FAMA study(3) (3% vs 2.5%), and higher than the FATA study(9) (3% *vs* 1.3%). The FAMA study(3) used a representative sample of the Portuguese population over 40 years old to study the prevalence of AF in 2010. The AF disease was determined using a 12-lead electrocardiogram. The FATA study(9) is an observational cross-sectional study from 2014 that included all patients aged 30 or above diagnosed with AF, enrolled in one of the eight Family Health Units of a northern region of Portugal. The higher values found in our study, when comparing with the FATA study,(9) corroborate our results: the prevalence of AF is increasing with time in the northern region of Portugal. As well as following expectations, this might also be related to the improvement of the coding process of the problems in the clinical process. It was found that the majority of AF patients in this study are elderly, as in the ATRIA study(4) (44.8% were aged 80 years or older *vs* 45% were aged 75 years or older).

The CHA_2DS_2-VASc scores found in our study are similar to those of FATA study(9): zero points (2.6% *vs* 2.3%), 1–3 points (40.7% *vs* 40.9%), 4–5 points (43.3% *vs* 41.6%), and ≥ 6 (13.4% *vs* 14.3%). In our study, 44,326 (72.9%) patients were adequately anticoagulated, which is an improvement relative to the FAMA (3), FATA (9), SAFIRA (10), and REACH (19) studies (38%, 56.8%, 43.7%, and 54% respectively). These studies have been published prior to the 2016 ESC guidelines (18). The guidelines advise that NOAC should be considered for men with a CHA_2DS_2-VASc score ≥ 1 and women with a score ≥ 2, which might have contributed to the improvement in anticoagulation in AF patients. It should be noted that in our study, 78.4% of patients with AF who are not anticoagulated have a high thrombotic risk (CHA_2DS_2-VASc score ≥ 3). This information is of most importance since clinicians should be proactive in the prevention of stroke in patients with AF.

In addition to evidence of anticoagulation undertreatment in AF patients, overtreatment is also found in AF patients who do not indicate anticoagulation. In our study, 40.9% of AF patients who are not indicated for anticoagulation are taking anticoagulants. Similar overtreatment rates occur in the REACH study,(19) where 43% of patients with AF and not indicated for anticoagulation were receiving anticoagulants. The FAMA,(3) FATA,(9) and SAFIRA(10) studies did not report overtreatment rates. A possible explanation for this overtreatment rate in our study might be a misuse of the CHA_2DS_2-VASc score. Some of these patients might also be prescribed an anticoagulation agent for other clinical reasons.

Although there has been a positive evolution regarding the number of patients with anticoagulated AF, warfarin remains the most prescribed anticoagulant. As noted before, this is not concordant with the ESC guidelines.(18) When analysing the distribution of drugs by CHA_2DS_2-VASc score and gender, it should be noted that in men who are not indicated to be anticoagulated (CHA_2DS_2-VASc score of zero), the most prescribed drug is rivaroxaban, which might result from another therapeutic indication, such as deep venous thrombosis. On the other hand, acenocoumarol appears more prescribed than dabigatran both in men with CHA_2DS_2-VASc scores of 7 and 8 and in women with a CHA_2DS_2-VASc score of 8. These results might be explained by the fact that clinicians prefer drugs with which they have more experience, such as

For peer review only - http://bmjopen.bmj.com/site/about/guidelines.xhtml

VKA in patients with a very high thrombotic risk. However, this does not follow the most current guidelines.

The SAFIRA(10) study showed a very high prevalence of cardiovascular risk factors in patients with AF. In Portugal, SCORE is recommended to be used for calculating cardiovascular risk in those between 40 and 65 years old.(16,17) However, we found no study that calculated cardiovascular risk by SCORE. The cardiovascular risk of patients aged 40–65 years and the total study population was considered, with a similar distribution, with most patients having a very high cardiovascular risk. The fact that the authors considered a very high cardiovascular risk, with no need to calculate SCORE, in patients with documented atherosclerotic cardiovascular disease, diabetes mellitus with at least one other cardiovascular risk factor or severe chronic kidney disease,(16) increased the number of cases with very high RCV if the only SCORE was used.

Implications for research and practic

The AF-React study made it possible to determine the prevalence of AF and to evaluate how patients with AF are being treated in northern Portugal. In Europe, there are no real-world studies with a large database of oral anticoagulation in patients with AF, so the AF-React study brings extremely relevant conclusions to Portugal and allows performing similar studies in Europe. For the future, it would be necessary for health authorities to allow continued access to these data sets, including mortality data, to draw conclusions (based on real-world) on the impact of the new treatments on patient-oriented outcomes.

For peer review only

FUNDING

This article was supported by National Funds through FCT - Fundação para a Ciência e a Tecnologia I.P., within CINTESIS, R&D Unit (reference UIDB/IC/4255/2020). The authors have received financial support of €4,400 EUR from the #H4A Primary Healthcare Research Network scholars program for support of research.

For peer review only

For peer review only - http://bmjopen.bmj.com/site/about/guidelines.xhtml

ETHICAL APPROVAL

The study protocol was approved by the Health Ethics Committee of the Regional Health Administration of Northern Portugal.

For peer review only

For peer review only - http://bmjopen.bmj.com/site/about/guidelines.xhtml

283 **COMPETING INTERESTS**

284 The authors declared no potential conflicts of interest with respect to the research,
285 authorship, and/or publication of this article.

286

287

For peer review only

For peer review only - http://bmjopen.bmj.com/site/about/guidelines.xhtml

ACKNOWLEDGEMENTS

A special thanks to all primary healthcare professionals who in their daily work in the Portuguese Health System fulfil the clinical records allowing researchers like us to achieve evidence that will help us all to improve the health of the Portuguese.

For peer review only

AUTHOR CONTRIBUTIONS

SSP and CM contributed to the conception of the work; all authors contributed to the design of the work. HM contributed to the data acquisition process; SPP, AT, and TH contributed to the analysis and interpretation of the work. SSP drafted the manuscript, HM, AT, TH, and CM critically revised the manuscript. All authors gave final approval and agreed to be accountable for all aspects of the work, ensuring integrity and accuracy.

For peer review only

For peer review only - http://bmjopen.bmj.com/site/about/guidelines.xhtml

WORD COUNT

2282 words

For peer review only

For peer review only - http://bmjopen.bmj.com/site/about/guidelines.xhtml

REFERENCES

1. Falk RH. Atrial fibrillation. N Engl J Med. 5 de Abril de 2001;344(14):1067–78.

2. Shafeeq H, Tran TH. New oral anticoagulants for atrial fibrillation: are they worth the risk? P T Peer-Rev J Formul Manag. Janeiro de 2014;39(1):54–64.

3. Bonhorst D, Mendes M, Adragão P, De Sousa J, Primo J, Leiria E, et al. Prevalence of atrial fibrillation in the Portuguese population aged 40 and over: the FAMA study. Rev Port Cardiol Orgao Of Soc Port Cardiol Port J Cardiol Off J Port Soc Cardiol. Março de 2010;29(3):331–50.

4. Ferreira RC, Macedo ME, Pinto F, Neves RC, Andrade C, Santos G. PROGRAMA NACIONAL PARA AS DOENÇAS CÉREBRO-CARDIOVASCULARES 2017 [Internet]. Direção Geral da Saúde; 2017.

5. da Silva RMFL. Novel oral anticoagulants in non-valvular atrial fibrillation. Cardiovasc Hematol Agents Med Chem. 2014;12(1):3–8.

6. Alonso A, Norby FL. Predicting Atrial Fibrillation and Its Complications. Circ J Off J Jpn Circ Soc. 25 de Abril de 2016;80(5):1061–6.

7. Aguilar MI, Hart R. Oral anticoagulants for preventing stroke in patients with non-valvular atrial fibrillation and no previous history of stroke or transient ischemic attacks. Cochrane Database Syst Rev. 20 de Julho de 2005;(3):CD001927.

8. Matchar DB, Samsa GP, Cohen SJ, Oddone EZ, Jurgelski AE. Improving the quality of anticoagulation of patients with atrial fibrillation in managed care organizations: results of the managing anticoagulation services trial. Am J Med. Julho de 2002;113(1):42–51.

9. Gomes E, Campos R, Morais R, Fernandes M. [FATA Study: prevalence of atrial fibrillation and antithrombotic therapy in primary health care in a northern city of Portugal]. Acta Med Port. Fevereiro de 2015;28(1):35–43.

10. Monteiro P. Estudo Safira: reflexões sobre a prevalência e os padrões de tratamento de fibrilhação auricular e risco cardiovascular em 7500 indivíduos com 65 ou mais anos. Rev Port Cardiol. Abril de 2018;37(4):307–13.

11. Connolly SJ, Ezekowitz MD, Yusuf S, Eikelboom J, Oldgren J, Parekh A, et al. Dabigatran versus warfarin in patients with atrial fibrillation. N Engl J Med. 17 de Setembro de 2009;361(12):1139–51.

12. Patel MR, Mahaffey KW, Garg J, Pan G, Singer DE, Hacke W, et al. Rivaroxaban versus warfarin in nonvalvular atrial fibrillation. N Engl J Med. 8 de Setembro de 2011;365(10):883–91.

13. Granger CB, Alexander JH, McMurray JJV, Lopes RD, Hylek EM, Hanna M, et al. Apixaban versus warfarin in patients with atrial fibrillation. N Engl J Med. 15 de Setembro de 2011;365(11):981–92.

14. Giugliano RP, Ruff CT, Braunwald E, Murphy SA, Wiviott SD, Halperin JL, et al. Edoxaban versus warfarin in patients with atrial fibrillation. N Engl J Med. 28 de Novembro de 2013;369(22):2093–104.

For peer review only

For peer review only - http://bmjopen.bmj.com/site/about/guidelines.xhtml

15. World Organization of National Colleges, Academies, and Academic Associations of General Practitioners/Family Physicians, editor. ICPC-2: international classification of primary care. 2nd ed. Oxford ; New York: Oxford University Press; 1998. 190 p. (Oxford medical publications).

16. European Association for Cardiovascular Prevention & Rehabilitation, Reiner Z, Catapano AL, De Backer G, Graham I, Taskinen M-R, et al. ESC/EAS Guidelines for the management of dyslipidaemias: the Task Force for the management of dyslipidaemias of the European Society of Cardiology (ESC) and the European Atherosclerosis Society (EAS). Eur Heart J. Julho de 2011;32(14):1769–818.

17. Authors/Task Force Members:, Piepoli MF, Hoes AW, Agewall S, Albus C, Brotons C, et al. 2016 European Guidelines on cardiovascular disease prevention in clinical practice: The Sixth Joint Task Force of the European Society of Cardiology and Other Societies on Cardiovascular Disease Prevention in Clinical Practice (constituted by representatives of 10 societies and by invited experts) Developed with the special contribution of the European Association for Cardiovascular Prevention & Rehabilitation (EACPR). Atherosclerosis. 2016;252:207–74.

18. Kirchhof P, Benussi S, Kotecha D, Ahlsson A, Atar D, Casadei B, et al. 2016 ESC Guidelines for the management of atrial fibrillation developed in collaboration with EACTS. Eur Heart J. 07 de 2016;37(38):2893–962.

19. Ruff CT, Bhatt DL, Steg PG, Gersh BJ, Alberts MJ, Hoffman EB, et al. Long-term cardiovascular outcomes in patients with atrial fibrillation and atherothrombosis in the REACH Registry. Int J Cardiol. 1 de Janeiro de 2014;170(3):413–8.

For peer review only

TABLES

Table 1. Characterization of the study population.

**Gender,** *n (%)*	
Female	33.437 (52.6%)
Male	30.089 (47.4%)
**Age (years),** $\bar{x} \pm s, min, máx$	76.49 ± 10.59, 18, 107
**Age groups,** *n(%)*	
<40 years	286 (0.5)
40-49 years	812 (1.3)
50-59 years	3286 (5.2)
60-69 years	10,089 (15.9)
70-79 years	20,602 (32.4)
≥ 80 years	28,451 (44.8)
**Professional situation,** *n(%)*	
Active	13,553 (21.3)
Not Active	4957 (7.8)
Student	54 (0.1)
Retired	44,685 (70.3)
Unknown	238 (0.4)
Not applicable	39 (0.1)
**Body Mass Index,** *n(%)*	9448 (14.9%)
**Body Mass Index,** *Med* $[Q_1; Q_3]$	29.1 [26.2; 32.6]
**Glomerular Filtration Rate** *n(%)*	
**Value available**	52,207 (82.2%)
**Missing record**	11,319 (17.8%)
**Stage of Chronic Kidney Disease**	
**1**	11,044 (21.2%)
**2**	19,693 (37.7%)
**3**	19,307 (37.0%)
**4**	2110 (4.0%)
**5**	53 (0.1%)
**Cardiovascular risk,** *n(%)*	
**Value available**	51,846 (81.6%)
**Missing record**	11,680 (18.4%)
**Classification of Cardiovascular Risk**	
**Low**	659 (1.3%)
**Moderate**	18,765 (36.2%)
**High**	2347 (4.5%)
**Very high**	30,075 (58.0%)
**CHA_2DS_2-VASc score,** *n(%)*	
**0 points**	1658 (2.6%)
**1-3 points**	25,843 (40.7%)
**4-5 points**	27,540 (43.3%)
**≥ 6 points**	8485 (13.4%)

For peer review only

Table 2. Prevalence (%) of atrial fibrillation in the northern region of Portugal between 2016 and 2018.

	< 40 years	40-49 years	50-59 years	60-69 years	70-79 years	≥ 80 years	Over 40 years
**2016**	0.019	0.092	0.435	1.611	5.264	10.572	2.295
**2017**	0.025	0.134	0.502	1.946	6.841	11.343	2.787
**2018**	0.030	0.138	0.620	2.333	6.693	13.047	3.044

For peer review only

Table 3. Medicated and non-medicated patients with and without a recommendation for anticoagulation.

Recommendation	Medicated, n (%)		Total
	Yes	No	
**Yes**	44,326 (69.8)	16,512 (26.0)	60,838 (95.8)
**No**	1100 (1.7)	1588 (2.5)	2688 (4.2)
**Total**	45,426 (71.5)	18,100 (28.5)	63,526 (100)

For peer review only

For peer review only - http://bmjopen.bmj.com/site/about/guidelines.xhtml

Table 4. Distribution of patients for CHA_2DS_2-VASc score value, gender, and respective drugs.

CHA_2DS_2-VASc score	Drugs, *n (%)*					
	**warfarin**	**rivaroxaban**	**apixaban**	**dabigatran**	**acenocoumarol**	**edoxaban**
Male						
**0**	127 (19.0)	248 (37.1)	134 (20.1)	88 (13.2)	41 (6.1)	30 (4.5)
**1**	426 (22.8)	623 (33.4)	344 (18.4)	276 (14.8)	129 (6.9)	70 (3.7)
**2**	1099 (26.7)	1125 (27.3)	801 (19.5)	590 (14.3)	369 (9.0)	132 (3.2)
**3**	1955 (31.1)	1531 (24.4)	1210 (19.3)	823 (13.1)	582 (9.3)	178 (2.8)
**4**	1636 (34.8)	1037 (22.1)	877 (18.7)	547 (11.6)	491 (10.4)	111 (2.4)
**5**	813 (33.9)	471 (19.7)	486 (20.3)	286 (11.9)	282 (11.8)	58 (2.4)
**6**	371 (36.6)	183 (18.0)	187 (18.4)	102 (10.1)	141 (13.9)	30 (3.0)
**7**	116 (36.3)	61 (19.1)	58 (18.1)	38 (11.9)	40 (12.5)	7 (2.2)
**8**	20 (37.7)	12 (22.6)	8 (15.1)	2 (3.8)	8 (15.1)	3 (5.7)
**9**	0 (0.0)	0 (0.0)	0 (0.0)	0 (0.0)	0 (0.0)	0 (0.0)
**Subtotal**	6563 (30.6)	5291 (24.7)	4105 (19.2)	2752 (12.9)	2083 (9.7)	619 (2.9)
Female						
**1**	125 (28.9)	118 (27.3)	88 (20.4)	49 (11.3)	35 (8.1)	17 (3.9)
**2**	432 (27.9)	418 (27.0)	345 (22.3)	189 (12.2)	110 (7.1)	52 (3.4)
**3**	1130 (29.3)	951 (24.7)	781 (20.3)	515 (13.4)	327 (8.5)	152 (3.9)
**4**	2195 (29.9)	1708 (23.2)	1565 (21.3)	979 (13.3)	694 (9.4)	208 (2.8)
**5**	2035 (33.6)	1287 (21.3)	1281 (21.2)	694 (11.5)	603 (10.0)	152 (2.5)
**6**	1024 (34.0)	627 (20.8)	623 (20.7)	330 (11.0)	322 (10.7)	86 (2.9)
**7**	398 (31.7)	232 (18.5)	312 (24.9)	150 (12.0)	135 (10.8)	28 (2.2)
**8**	148 (32.7)	85 (18.8)	121 (26.8)	43 (9.5)	47 (10.4)	8 (1.8)
**9**	23 (39.0)	11 (18.6)	14 (23.7)	6 (10.2)	3 (5.1)	2 (3.4)
**Subtotal**	7510 (31.3)	5437 (22.6)	5130 (21.4)	2955 (12.3)	2276 (9.5)	705 (2.9)
**Total**	31%	23.6%	20.3%	12.6%	9.6%	2.9%

For peer review only

Table 5. Pathologies by the International Classification of Primary Care – 2nd Edition.

Pathologies	n (%)
chronic alcohol abuse (P15)	2392 (3.8)
tobacco abuse (P17)	2880 (4.5)
lipid disorder (T93)	33,095 (52.1)
atherosclerosis/PVD (K92)	4617 (7.3)
diabetes insulin dependente (T89 + T90)	1551 (2.4)
diabetes non-insulin dependente (T90)	16,553 (26.1)
coronary heart disease ischaemic heart disease w. angina (K74)	3370 (5.3)
ischaemic heart disease w/o angina (K76)	3185 (5.0)
acute myocardial infarction (K75)	2450 (3.9)
hypertension complicated (K87)	17,598 (27.7)
hypertension uncomplicated (K86)	31,466 (49.5)
heart failure (K77)	17,530 (27.6)

For peer review only

For peer review only - http://bmjopen.bmj.com/site/about/guidelines.xhtml

STROBE Statement—Checklist of items that should be included in reports of ***cohort studies***

	Item No	Recommendation	Page No
**Title and abstract**	1	(*a*) Indicate the study's design with a commonly used term in the title or the abstract	2
		(*b*) Provide in the abstract an informative and balanced summary of what was done and what was found	2
**Introduction**			
Background/rationale	2	Explain the scientific background and rationale for the investigation being reported	4
Objectives	3	State specific objectives, including any prespecified hypotheses	4
**Methods**			
Study design	4	Present key elements of study design early in the paper	5
Setting	5	Describe the setting, locations, and relevant dates, including periods of recruitment, exposure, follow-up, and data collection	5
Participants	6	(*a*) Give the eligibility criteria, and the sources and methods of selection of participants. Describe methods of follow-up<br>(*b*) For matched studies, give matching criteria and number of exposed and unexposed	5
Variables	7	Clearly define all outcomes, exposures, predictors, potential confounders, and effect modifiers. Give diagnostic criteria, if applicable	5
Data sources/ measurement	8*	For each variable of interest, give sources of data and details of methods of assessment (measurement). Describe comparability of assessment methods if there is more than one group	5
Bias	9	Describe any efforts to address potential sources of bias	NA
Study size	10	Explain how the study size was arrived at	5
Quantitative variables	11	Explain how quantitative variables were handled in the analyses. If applicable, describe which groupings were chosen and why	5
Statistical methods	12	(*a*) Describe all statistical methods, including those used to control for confounding<br>(*b*) Describe any methods used to examine subgroups and interactions<br>(*c*) Explain how missing data were addressed<br>(*d*) If applicable, explain how loss to follow-up was addressed<br>(*e*) Describe any sensitivity analyses	5
**Results**			
Participants	13*	(a) Report numbers of individuals at each stage of study—eg numbers potentially eligible, examined for eligibility, confirmed eligible, included in the study, completing follow-up, and analysed<br>(b) Give reasons for non-participation at each stage<br>(c) Consider use of a flow diagram	6
Descriptive data	14*	(a) Give characteristics of study participants (eg demographic, clinical, social) and information on exposures and potential confounders<br>(b) Indicate number of participants with missing data for each variable of interest<br>(c) Summarise follow-up time (eg, average and total amount)	6, 17
Outcome data	15*	Report numbers of outcome events or summary measures over time	6, 17

For peer review only

For peer review only - http://bmjopen.bmj.com/site/about/guidelines.xhtml

Main results	16	(*a*) Give unadjusted estimates and, if applicable, confounder-adjusted estimates and their precision (eg, 95% confidence interval). Make clear which confounders were adjusted for and why they were included<br>(*b*) Report category boundaries when continuous variables were categorized<br>(*c*) If relevant, consider translating estimates of relative risk into absolute risk for a meaningful time period	6, 20, 21
Other analyses	17	Report other analyses done—eg analyses of subgroups and interactions, and sensitivity analyses	6, 18, 19, 20
**Discussion**			
Key results	18	Summarise key results with reference to study objectives	7
Limitations	19	Discuss limitations of the study, taking into account sources of potential bias or imprecision. Discuss both direction and magnitude of any potential bias	3
Interpretation	20	Give a cautious overall interpretation of results considering objectives, limitations, multiplicity of analyses, results from similar studies, and other relevant evidence	7-8
Generalisability	21	Discuss the generalisability (external validity) of the study results	7-8
**Other information**			
Funding	22	Give the source of funding and the role of the funders for the present study and, if applicable, for the original study on which the present article is based	9

*Give information separately for exposed and unexposed groups.

Note: An Explanation and Elaboration article discusses each checklist item and gives methodological background and published examples of transparent reporting. The STROBE checklist is best used in conjunction with this article (freely available on the Web sites of PLoS Medicine at http://www.plosmedicine.org/, Annals of Internal Medicine at http://www.annals.org/, and Epidemiology at http://www.epidem.com/). Information on the STROBE Initiative is available at http://www.strobe-statement.org.

For peer review only

For peer review only - http://bmjopen.bmj.com/site/about/guidelines.xhtml

BMJ Open

AF-React study – Atrial fibrillation management strategies in clinical practice: retrospective longitudinal study from real-world data in Northern Portugal

Journal:	*BMJ Open*
Manuscript ID	bmjopen-2020-040404.R1
Article Type:	Original research
Date Submitted by the Author:	26-Oct-2020
Complete List of Authors:	Silva Pinto, Susana; University of Porto Faculty of Medicine, Family Medicine Unit, Department of Community Medicine, Information and Health Decision Sciences (MEDCIDS); Center for Health Technology and Services Research<br>Teixeira, Andreia; University of Porto Faculty of Medicine, Department of Community Medicine, Information and Health Decision Sciences (MEDCIDS); Center for Health Technology and Services Research<br>Henriques, Teresa; University of Porto Faculty of Medicine, Department of Community Medicine, Information and Health Decision Sciences (MEDCIDS); Center for Health Technology and Services Research<br>Monteiro, Hugo; Research and Planning Department, Regional Health Administration of Northern, Min. of Health Portugal<br>Martins, Carlos; University of Porto Faculty of Medicine, Family Medicine Unit, Department of Community Medicine, Information and Health Decision Sciences (MEDCIDS); Center for Health Technology and Services Research
**Primary Subject Heading**:	Cardiovascular medicine
Secondary Subject Heading:	Epidemiology
Keywords:	PRIMARY CARE, STROKE MEDICINE, Adult cardiology < CARDIOLOGY

SCHOLARONE™
Manuscripts

I, the Submitting Author has the right to grant and does grant on behalf of all authors of the Work (as defined in the below author licence), an exclusive licence and/or a non-exclusive licence for contributions from authors who are: i) UK Crown employees; ii) where BMJ has agreed a CC-BY licence shall apply, and/or iii) in accordance with the terms applicable for US Federal Government officers or employees acting as part of their official duties; on a worldwide, perpetual, irrevocable, royalty-free basis to BMJ Publishing Group Ltd ("BMJ") its licensees and where the relevant Journal is co-owned by BMJ to the co-owners of the Journal, to publish the Work in this journal and any other BMJ products and to exploit all rights, as set out in our licence.

The Submitting Author accepts and understands that any supply made under these terms is made by BMJ to the Submitting Author unless you are acting as an employee on behalf of your employer or a postgraduate student of an affiliated institution which is paying any applicable article publishing charge ("APC") for Open Access articles. Where the Submitting Author wishes to make the Work available on an Open Access basis (and intends to pay the relevant APC), the terms of reuse of such Open Access shall be governed by a Creative Commons licence – details of these licences and which Creative Commons licence will apply to this Work are set out in our licence referred to above.

Other than as permitted in any relevant BMJ Author's Self Archiving Policies, I confirm this Work has not been accepted for publication elsewhere, is not being considered for publication elsewhere and does not duplicate material already published. I confirm all authors consent to publication of this Work and authorise the granting of this licence.

For peer review only

TITLE PAGE

AF-React study – Atrial fibrillation management strategies in clinical practice: retrospective longitudinal study from real-world data in Northern Portugal

Pinto, Susana Silva;[1, 2,3] Email: susyapinto@gmail.com
[1]ACeS Santo Tirso/Trofa, Regional Health Administration of Northern, Min. of Health Portugal.
[2]Department of Community Medicine, Information and Health Decision Sciences (MEDCIDS), Faculty of Medicine, University of Porto, Porto, Portugal.
[3]Centre for Health Technology and Services Research (CINTESIS), University of Porto, Porto, Portugal.

Teixeira, Andreia;[2, 3, 4] Email: andreiasofiat@hotmail.com
[2]Department of Community Medicine, Information and Health Decision Sciences (MEDCIDS), Faculty of Medicine, University of Porto, Porto, Portugal.
[3]Centre for Health Technology and Services Research (CINTESIS), University of Porto, Porto, Portugal.
[4]ARC4DigiT, Polytechnic Institute of Viana do Castelo, Portugal.

Henriques, Teresa S.;[2, 3] Email: teresasarhen@gmail.com
[2]Department of Community Medicine, Information and Health Decision Sciences (MEDCIDS), Faculty of Medicine, University of Porto, Porto, Portugal.
[3]Centre for Health Technology and Services Research (CINTESIS), University of Porto, Porto, Portugal.

Monteiro, Hugo;[5] Email: hfmonteiro@arsnorte.min-saude.pt
[5]Research and Planning Department, Regional Health Administration of Northern, Min. of Health Portugal.

Martins, Carlos.[2, 3] Email: carlosmartins20@gmail.com
[2]Department of Community Medicine, Information and Health Decision Sciences (MEDCIDS), Faculty of Medicine, University of Porto, Porto, Portugal.
[3]Centre for Health Technology and Services Research (CINTESIS), University of Porto, Porto, Portugal.

Corresponding Author
Susana Silva Pinto
susyapinto@gmail.com

For peer review only

ABSTRACT

Objectives

To determine the prevalence of AF and to assess how these patients are being cared for: what anticoagulants are being prescribed and are they being prescribed as recommended?

Design

Retrospective longitudinal study.

Setting

This study was conducted in Regional Health Administration of Northern Portugal.

Participants

This study using a database that included 63,526 patients with code K78 of the International Classification of Primary Care between January 2016 and December 2018.

Results

The prevalence of AF among adults over 40 years in the northern region of Portugal was 2.3% in 2016, 2.8% in 2017, and 3% in 2018. From a total of 63,526 patients, 95.8% had an indication to receive anticoagulation therapy. Of these, 44,326 (72.9%) are being treated with anticoagulants: 17,936 (40.5%) were prescribed with vitamin K antagonists and 26,390 (59.5%) with non-vitamin K antagonist anticoagulants.

Conclusions

The prevalence of AF is 3%. Here we report evidence of undertreatment, but also of overtreatment. Albeit having an indication, there is a considerable proportion of patients (27.1%) that are not anticoagulated, and among AF patients without indication to receive anticoagulation therapy, there is a considerable proportion (40.9%) receiving anticoagulants. The AF-React study brings extremely relevant conclusions to Portugal and is following real-world studies in patients with AF in Europe, presenting some data not studied yet.

Keywords

atrial fibrillation, anticoagulants, prevention, stroke; primary health care.

Strengths and limitations of this study

- The main limitation of this retrospective study is that the AF assessment is based on data coded in the clinical process in Primary Health Care.
- There might be more patients in the northern region of Portugal with AF, but they are not coded at Primary Health Care and, therefore, were not included in this study.
- The lack of ICPC-2 coding for problems such as mechanical prosthesis valve and rheumatic moderate or severe mitral stenosis might be another limitation.
- There might be a small percentage of patients well medicated with vitamin K antagonists because they are in one of those situations.
- Few of the 63,526 patients will be diagnosed with atrial flutter, however for patients with atrial flutter, anticoagulant therapy is recommended according to the same risk profile used for AF.

For peer review only - http://bmjopen.bmj.com/site/about/guidelines.xhtml

BACKGROUND

Atrial fibrillation (AF) is the most common type of sustained cardiac arrhythmia.(1) The clinical relevance of AF is associated to the loss of effective atrial contractility resulting in deficient emptying of the left atrial appendage. This process increases the risk of thrombus formation and thromboembolic events.(1)

Due to the ageing of the population, the number of people affected by AF worldwide is projected to exceed 12 million by 2050.(2) In Portugal in 2010, the FAMA study(3) verified an overall prevalence of AF of 2.5% in a population sample older than 40 years, with a significant increase after the age of 70 years.(3)

In Portugal, despite a considerable reduction in ischemic stroke mortality below 70 years (39% reduction) in 2015 compared to 2011, stroke remains the main cause of mortality related to vascular diseases.(4) Patients with AF have a five-fold increased risk of stroke.(5) This arrhythmia is currently responsible for about 15% of stroke cases.(3) Stroke is a major complication associated with AF, which contributes to the morbidity and mortality associated with the disease.(6)

Previous studies have reported that oral anticoagulation with vitamin K antagonists (VKA), as warfarin, reduces stroke and mortality in AF patients.(7) The non-vitamin K antagonist oral anticoagulants (NOAC) – dabigatran, rivaroxaban, apixaban, edoxaban – have been shown to be superior to warfarin in the prevention of thromboembolic events in patients with non-valvular AF, providing increased safety and an overall reduction in the number of cases of bleeding events(8–11). The cost and morbidity associated with stroke and intracranial haemorrhage are high, so the lower prevalence of these events with NOACs, compared to warfarin, is one factor that might help this drug to become cost-effective relative to warfarin.(12) Furthermore, according to the Portuguese Directorate-General of Health, the reduction in ischemic stroke mortality below 70 years was due to the introduction into clinical practice of NOAC as an antithrombotic therapy for AF. (4)

In a cross-sectional study of patients with AF done in eight Family Health Units of Northern Portugal (the FATA study),(13) only 56.8% of patients with AF were prescribed adequate oral anticoagulation according to the recommendations of the European Society of Cardiology (ESC) Guidelines in 2010. In the SAFIRA study,(14) 56.3% of patients with AF were not anticoagulated.

There are no randomised controlled trials (RCT) directly comparing NOACs to each other (or to placebo). All RCTs compared a NOAC to warfarin. For example, the RE-LY,(8) ROCKET-AF,(9) ARISTOLETE,(10) and ENGAGE AF-TIMI 48(11) studies included 18,113, 14,264, 18,201 and 21,105 patients with AF respectively. In fact, these trials were crucial for the efficacy and safety evaluation of these drugs and their approval. However, the real-world studies might contribute with additional relevant evidence because they include epidemiological data and drug prescribing patterns for a larger number of patients in the context of daily clinical practice. In Portugal there are no real-world studies with a large database of oral anticoagulation in patients with AF.

In Europe, there are some study with a large database: A study involving databases from 6 European countries concluded that in overall, apixaban and rivaroxaban increased its use during the study period while dabigatran decreased and there was variability in patient characteristics such as comorbidities, potentially interacting drugs and dose adjustment.(15) Komen et al (2017) investigate the influence of patient characteristics such as age and stroke and bleeding risks on decisions for antithrombotic treatment in patients with AF and concluded that apixaban was favored for elderly and high-risk patients whereas dabigatran was used in lower risk patients.(16) Mueller et al (2019) compare the clinical effectiveness and safety of

For peer review only

For peer review only - http://bmjopen.bmj.com/site/about/guidelines.xhtml

NOACs in patients with AF in routine clinical practice. All NOACs were similarly effective in preventing strokes and systemic embolisms, while patients being treated with rivaroxaban exhibited the highest bleeding risks.(17)

The AF-React study aims to determine the prevalence of AF in the northern region of Portugal and to assess how patients with AF are being cared for: what anticoagulants are being prescribed and are they being prescribed as recommended?

For peer review only - http://bmjopen.bmj.com/site/about/guidelines.xhtml

METHOD

This retrospective longitudinal study was conducted using a Regional Health Administration database of Northern Portugal. We consider the code for AF, K78, of the International Classification of Primary Care (ICPC-2)(15). Therefore, we included all the adults (age ≥ 18 years) with the code K78 as an active problem between January 2016 and December 2018. The database included clinical information from 63,526 patients with AF. For each patient, the clinical information collected was the last available between January 2016 and December 2018.

The prevalence of AF was calculated based on the age pyramid of the population enrolled in Regional Health Administration of Northern Portugal in 2016, 2017, and 2018.

The CV risk (cardiovascular risk) was defined in four stages based on SCORE (Systematic Coronary Risk Evaluation), as low, moderate, high, and very high, if less than 1%, between 1% and 5%, between 5% and 10%, and greater than or equal to 10%, respectively, for patients between 40 and 65 years old. The percentage SCORE value was extracted from the clinical process. The high stage was also considered both in patients with diabetes and no other CV risk factor or target organ lesion or in patients with moderate CKD (chronic kidney disease).(18,19) The very high stage was also considered in patients with documented atherosclerotic cardiovascular disease, patients with diabetes and another CV risk factor, or target organ lesion or in patients with severe CKD, or any combination of these.(18,19) The stages of CKD were defined based on the glomerular filtration rate (GFR): stage 1, GFR ≥ 90 ml/min; stage 2, GFR]90-60] ml/min; stage 3, GFR]60-30] ml/min, stage 4, GFR [30-15] ml/min; and stage 5, GFR < 15ml/min. The high and very high stages CV risk were based on the codification of health problems by ICPC-2 and the patient's GFR registered in the clinical process.

The CHA_2DS_2-VASc score was calculated according to the ESC guidelines in 2016 for AF(20), as follows by ICPC-2 coding (21): C-Congestive heart failure (K77): 1 point; H-Hypertension (K86 or K87): 1 point; A_2-Age > 75 years or older: 2 points; D-Diabetes mellitus (T89 or T90): 1 point; S_2-Stroke (K89, K90 or K91): 2 points; V-Vascular disease (K75 or K92): 1 point; A-Age 65-74 years: 1 point; Sc-Sex category (female): 1 point. Oral anticoagulation was considered in a male with a CHA_2DS_2-VASc score ≥ 1 point and in a female with a score ≥ 2 points.

The categorical variables were described using absolute and relative frequencies, n (%). The normally distributed continuous variables were described by the mean and respective standard deviation, $\bar{x} \pm s$, and by the minimum (min) and maximum (max) values. In the case of continuous variables not normally distributed, the data are presented by the median and respective interquartile range, Med [Q_1; Q_3], where Q_1 is the first quartile and Q_3 is the third quartile. The normality was verified by observing the histograms.

The database was exported to Microsoft Excel 2016®, and the statistical analysis was performed using SPSS Statistics 25.0® and software R. Data were obtained with specific filters, without aggregation at the individual level for general characterisation analysis.

The data processing was carried out through the Department of Studies and Planning of the Regional Health Administration of Northern Portugal (Ministry of Health, Portugal). The data was extracted from the server through an anonymised data processing and editing platform and delivered securely (and in accordance with legal regulations and due approval) to the principal investigator. Patients and public were not involved in the development of the research question.

For peer review only - http://bmjopen.bmj.com/site/about/guidelines.xhtml

RESULTS

A total of 63,526 patients were identified to have the diagnosis of atrial fibrillation/flutter (ICPC-2, K78 code) in the northern region of Portugal. The average age was 76 ± 10.6 years (range, from 18 to 107 years) and 53% were female. Table 1 provides a detailed characterisation of the study population. Considering a total of adults over 40 years included in the Regional Health Administration of Northern Portugal, the prevalence of AF among the adults over 40 years in the northern region of Portugal was 2.3% in 2016, 2.8% in 2017, and 3% in 2018 (Table 2).

From a total of 63,526 patients, 60,838 (95.8%) had an indication to receive anticoagulation therapy: male with CHA_2DS_2-VASc score ≥ 1 and female with CHA_2DS_2-VASc score ≥ 2. Among these 60,838 patients, 16,512 (27,1%) were not being treated with anticoagulants, and 79% of those 16,512 have a CHA_2DS_2-VASc score of 3 or higher.

Among the 44,326 being treated with anticoagulants, 17,936 (40.5%) were prescribed with VKAs (warfarin and acenocoumarol) and 26,390 (59.5%) with NOACs (dabigatran, rivaroxaban, apixaban and edoxaban). The most commonly prescribed drug in medicated patients is warfarin (31%), followed by rivaroxaban (23.6%), apixaban (20.3%), dabigatran (12.6%), acenocoumarol (9.6%), and edoxaban (2.9%).

The CHA_2DS_2-VASc score has the following distribution: zero points (2.6%), 1–3 points (40.7%), 4–5 points (43.3%), and ≥ 6 points (13.4%). From the total of 30,089 males, 1658 (5.5%) had a CHA_2DS_2-VASc score of zero, and from a total of 33,437 females, 1030 (3.1%) had a CHA_2DS_2-VASc score of 1. This means that these 2688 (4.2%) patients of the total had no indication to receive anticoagulation therapy. Of these 2688 patients, 1100 were receiving anticoagulants (Table 3).

The distributions of the different drugs by CHA_2DS_2-VASc score and gender are shown in Table 4. For males, rivaroxaban (37.1%) and apixaban (20.1%) are more often prescribed than warfarin (19%) in the group with a CHA_2DS_2-VASc score of zero. In particular, apixaban is more prescribed than rivaroxaban in groups with CHA_2DS_2-VASc scores of 5 or 6 (20.3% *vs* 19.7% and 18.4% *vs* 18%, respectively). For females, apixaban is more prescribed than rivaroxaban in the groups with CHA_2DS_2-VASc scores of 7, 8, or 9 (24.9% *vs* 18.5%, 26.8% *vs* 18.8%, and 23.7% *vs* 18.6%, respectively).

The CV risk was available for 6531 (10.3%) of the patients between 40 and 65 years old. The CV risk values, for patients in that age group, were distributed as follows: low - 1.0%, moderate - 40.3%, high - 3.0% and very high - 46.8%.

The glomerular filtration rate was available for 52,207 patients (82.2%) and followed this distribution: stage 1 (21.2%); stage 2 (37.7%); stage 3 (37.0%); stage 4 (4.0%); stage 5 (0.1%). The VKAs (warfarin and acenocoumarol) were more prescribed than NOACs (dabigatran, rivaroxaban, apixaban and edoxaban) in stages 4 and 5. The distribution of patients for CKD' stages by respective drugs is represented in Table 5.

From 63,526 patients with AF, between 2016 and 2018, 17,936 (28.2%) were always prescribed with VKA, 21,854 (34.4%) were always prescribed with NOAC, 3508 (5.5%) were prescribed VKA who switched to NOAC, and 1504 (2.4%) were prescribed with NOAC who switched to another NOAC.

The prevalence of each of the comorbidities studied in patients with AF is represented in Table 6. We found that the most prevalent comorbidities associated with AF are hypertension (77.2%), dyslipidemia (52.1%), diabetes (28.5%), and heart failure (27.6%).

For peer review only

DISCUSSION

Summary

In the AF-React study, a prevalence of AF was 3% in 2018. A considerable proportion of patients, with indication for anticoagulation therapy, are not anticoagulated (27.1%), and among AF patients without indication to receive anticoagulation therapy, there is a considerable proportion (40.9%) receiving anticoagulants.

Comparison with existing literature

The prevalence of AF found in this study is similar to that of the FAMA study(3) (3% vs 2.5%), and higher than the FATA study(13) (3% *vs* 1.3%). The FAMA study(3) used a representative sample of the Portuguese population over 40 years old to study the prevalence of AF in 2010. The AF disease was determined using a 12-lead electrocardiogram. The FATA study(13) is an observational cross-sectional study from 2014 that included all patients aged 30 or above diagnosed with AF, enrolled in one of the eight Family Health Units of a northern region of Portugal. The higher values found in our study, when comparing with the FATA study,(13) corroborate our results: the prevalence of AF is increasing with time in the northern region of Portugal. As well as following expectations, this might also be related to the improvement of the coding process of the problems in the clinical process. It was found that the majority of AF patients in this study are elderly, as in the ATRIA study(4) (44.8% were aged 80 years or older *vs* 45% were aged 75 years or older).

The CHA_2DS_2-VASc scores found in our study are similar to those of FATA study(13): zero points (2.6% *vs* 2.3%), 1–3 points (40.7% *vs* 40.9%), 4–5 points (43.3% *vs* 41.6%), and ≥ 6 (13.4% *vs* 14.3%). In our study, 44,326 (72.9%) patients were adequately anticoagulated, which is an improvement relative to the FAMA (3), FATA (13), SAFIRA (14), and REACH (22) studies (38%, 56.8%, 43.7%, and 54% respectively). These studies have been published prior to the 2016 ESC guidelines (20). The guidelines advise that NOAC should be considered for men with a CHA_2DS_2-VASc score ≥ 1 and women with a score ≥ 2, which might have contributed to the improvement in anticoagulation in AF patients. It should be noted that in our study, 79% of patients with AF who are not anticoagulated have a high thrombotic risk (CHA_2DS_2-VASc score ≥ 3). This information is of most importance since clinicians should be proactive in the prevention of stroke in patients with AF.

In addition to evidence of anticoagulation undertreatment in AF patients, overtreatment is also found in AF patients who do not indicate anticoagulation. In our study, 40.9% of AF patients who are not indicated for anticoagulation are taking anticoagulants. Similar overtreatment rates occur in the REACH study,(22) where 43% of patients with AF and not indicated for anticoagulation were receiving anticoagulants. The FAMA,(3) FATA,(13) and SAFIRA(14) studies did not report overtreatment rates. A possible explanation for this overtreatment rate in our study might be a misuse of the CHA_2DS_2-VASc score. Some of these patients might also be prescribed an anticoagulation agent for other clinical reasons.

Although there has been a positive evolution regarding the number of patients with anticoagulated AF, warfarin remains the most prescribed anticoagulant. As noted before, this is not concordant with the 2016 ESC guidelines.(20) When analysing the distribution of drugs by CHA_2DS_2-VASc score and gender, it should be noted that in men who are not indicated to be anticoagulated (CHA_2DS_2-VASc score of zero), the most prescribed drug is rivaroxaban, which might result from another therapeutic indication, such as deep venous thrombosis. On the other hand, acenocoumarol appears more prescribed than dabigatran both in men with CHA_2DS_2-VASc scores of 7 and 8 and in women with a CHA_2DS_2-VASc score of 8. These results might be explained by the fact that clinicians prefer drugs with which they have more experience, such as

For peer review only - http://bmjopen.bmj.com/site/about/guidelines.xhtml

VKA in patients with a very high thrombotic risk. However, this does not follow the most current guidelines.

The SAFIRA(14) study showed a very high prevalence of CV risk factors in patients with AF. In Portugal, SCORE is recommended to be used for calculating CV risk in those between 40 and 65 years old.(18,19) However, we found no study that calculated CV risk by SCORE. The CV risk of patients aged 40–65 years was considered, with most patients having a very high CV risk. The fact that the authors considered a very high CV risk, with no need to calculate SCORE, in patients with documented atherosclerotic cardiovascular disease, diabetes mellitus with at least one other CV risk factor or severe chronic kidney disease,(18) increased the number of cases with very high CV risk if the only SCORE was used.

The SAFIRA(14) study was found that 24.7% of the patients were incorrectly medicated (inadequate dose, inadequate number of doses or creatinine clearance incompatible with the use of NOAC), but it does not discriminate patients by stages of CKD. The AF-React study shows that NOACs are being prescribed in stage 5 of CKD, and this is contraindicated by the guidelines in force at the date of prescription (between 2016 to 2018).(20,23) In stage 4 of CKD, 2016 ESC Guidelines(20) does not recommend the use of NOACs and EHRA practical guide(23) recommends dose adjustment for apixaban, rivaroxaban and edoxaban and does not recommend the use of dabigatran. This data opens the door to a more detailed analysis in the future about the prescriptions in stage 4.

Implications for research and practic

The AF-React study made it possible to determine the prevalence of AF and to evaluate how patients with AF are being treated in northern Portugal, so the AF-React study brings extremely relevant conclusions to Portugal and is following real-world studies in patients with AF in Europe, presenting some data not studied yet: distribution of patients for CHA_2DS_2-VASc score value, gender and respective drugs, classification of CV risk and distribution of patients for Chronic Kidney Disease' stages by respective drugs. For the future, it would be necessary for health authorities to allow continued access to these data sets, including mortality data, to draw conclusions (based on real-world) on the impact of the new treatments on patient-oriented outcomes.

For peer review only

FUNDING

This article was supported by National Funds through FCT - Fundação para a Ciência e a Tecnologia I.P., within CINTESIS, R&D Unit (reference UIDB/IC/4255/2020). The authors have received financial support of €4,400 EUR from the #H4A Primary Healthcare Research Network scholars program for support of research.

For peer review only

For peer review only - http://bmjopen.bmj.com/site/about/guidelines.xhtml

ETHICAL APPROVAL

The study protocol was approved by the Health Ethics Committee of the Regional Health Administration of Northern Portugal.

For peer review only

For peer review only - http://bmjopen.bmj.com/site/about/guidelines.xhtml

COMPETING INTERESTS

The authors declared no potential conflicts of interest with respect to the research, authorship, and/or publication of this article.

For peer review only - http://bmjopen.bmj.com/site/about/guidelines.xhtml

ACKNOWLEDGEMENTS

A special thanks to all primary healthcare professionals who in their daily work in the Portuguese Health System fulfil the clinical records allowing researchers like us to achieve evidence that will help us all to improve the health of the Portuguese.

For peer review only

AUTHOR CONTRIBUTIONS

SSP and CM contributed to the conception of the work; all authors contributed to the design of the work. HM contributed to the data acquisition process; SPP, AT, and TH contributed to the analysis and interpretation of the work. SSP drafted the manuscript, HM, AT, TH, and CM critically revised the manuscript. All authors gave final approval and agreed to be accountable for all aspects of the work, ensuring integrity and accuracy.

For peer review only

WORD COUNT

2811 words

For peer review only

For peer review only - http://bmjopen.bmj.com/site/about/guidelines.xhtml

DATA SHARING STATEMENT

Data are available upon reasonable request.

For peer review only

For peer review only - http://bmjopen.bmj.com/site/about/guidelines.xhtml

REFERENCES

1. Falk RH. Atrial fibrillation. N Engl J Med. 5 april 2001;344(14):1067–78.

2. Shafeeq H, Tran TH. New oral anticoagulants for atrial fibrillation: are they worth the risk? P T Peer-Rev J Formul Manag. jan 2014;39(1):54–64.

3. Bonhorst D, Mendes M, Adragão P, De Sousa J, Primo J, Leiria E, et al. Prevalence of atrial fibrillation in the Portuguese population aged 40 and over: the FAMA study. Rev Port Cardiol Orgao Of Soc Port Cardiol Port J Cardiol Off J Port Soc Cardiol. Março de 2010;29(3):331–50.

4. Ferreira RC, Macedo ME, Pinto F, Neves RC, Andrade C, Santos G. PROGRAMA NACIONAL PARA AS DOENÇAS CÉREBRO-CARDIOVASCULARES 2017 [Internet]. Direção Geral da Saúde; 2017.

5. da Silva RMFL. Novel oral anticoagulants in non-valvular atrial fibrillation. Cardiovasc Hematol Agents Med Chem. 2014;12(1):3–8.

6. Alonso A, Norby FL. Predicting Atrial Fibrillation and Its Complications. Circ J Off J Jpn Circ Soc. 25 april 2016;80(5):1061–6.

7. Aguilar MI, Hart R. Oral anticoagulants for preventing stroke in patients with non-valvular atrial fibrillation and no previous history of stroke or transient ischemic attacks. Cochrane Database Syst Rev. 20 July 2005;(3):CD001927.

8. Connolly SJ, Ezekowitz MD, Yusuf S, Eikelboom J, Oldgren J, Parekh A, et al. Dabigatran versus warfarin in patients with atrial fibrillation. N Engl J Med. 17 september 2009;361(12):1139–51.

9. Patel MR, Mahaffey KW, Garg J, Pan G, Singer DE, Hacke W, et al. Rivaroxaban versus warfarin in nonvalvular atrial fibrillation. N Engl J Med. 8 september 2011;365(10):883–91.

10. Granger CB, Alexander JH, McMurray JJV, Lopes RD, Hylek EM, Hanna M, et al. Apixaban versus warfarin in patients with atrial fibrillation. N Engl J Med. 15 september 2011;365(11):981–92.

11. Giugliano RP, Ruff CT, Braunwald E, Murphy SA, Wiviott SD, Halperin JL, et al. Edoxaban versus warfarin in patients with atrial fibrillation. N Engl J Med. 28 november 2013;369(22):2093–104.

12. Matchar DB, Samsa GP, Cohen SJ, Oddone EZ, Jurgelski AE. Improving the quality of anticoagulation of patients with atrial fibrillation in managed care organizations: results of the managing anticoagulation services trial. Am J Med. July 2002;113(1):42–51.

13. Gomes E, Campos R, Morais R, Fernandes M. [FATA Study: prevalence of atrial fibrillation and antithrombotic therapy in primary health care in a northern city of Portugal]. Acta Med Port. February 2015;28(1):35–43.

14. Monteiro P. Estudo Safira: reflexões sobre a prevalência e os padrões de tratamento de fibrilhação auricular e risco cardiovascular em 7500 indivíduos com 65 ou mais anos. Rev Port Cardiol. Abril 2018;37(4):307–13.

For peer review only - http://bmjopen.bmj.com/site/about/guidelines.xhtml

15. Ibáñez L, Sabaté M, Vidal X, Ballarin E, Rottenkolber M, Schmiedl S, et al. Incidence of direct oral anticoagulant use in patients with nonvalvular atrial fibrillation and characteristics of users in 6 European countries (2008–2015): A cross-national drug utilization study. Br J Clin Pharmacol. November 2019;85(11):2524–39.

16. Komen J, Forslund T, Hjemdahl P, Wettermark B. Factors associated with antithrombotic treatment decisions for stroke prevention in atrial fibrillation in the Stockholm region after the introduction of NOACs. Eur J Clin Pharmacol. October 2017;73(10):1315–22.

17. Mueller T, Alvarez-Madrazo S, Robertson C, Wu O, Bennie M. Comparative safety and effectiveness of direct oral anticoagulants in patients with atrial fibrillation in clinical practice in Scotland. Br J Clin Pharmacol. February 2019;85(2):422–31.

18. European Association for Cardiovascular Prevention & Rehabilitation, Reiner Z, Catapano AL, De Backer G, Graham I, Taskinen M-R, et al. ESC/EAS Guidelines for the management of dyslipidaemias: the Task Force for the management of dyslipidaemias of the European Society of Cardiology (ESC) and the European Atherosclerosis Society (EAS). Eur Heart J. July 2011;32(14):1769–818.

19. Authors/Task Force Members:, Piepoli MF, Hoes AW, Agewall S, Albus C, Brotons C, et al. 2016 European Guidelines on cardiovascular disease prevention in clinical practice: The Sixth Joint Task Force of the European Society of Cardiology and Other Societies on Cardiovascular Disease Prevention in Clinical Practice (constituted by representatives of 10 societies and by invited experts) Developed with the special contribution of the European Association for Cardiovascular Prevention & Rehabilitation (EACPR). Atherosclerosis. 2016;252:207–74.

20. Kirchhof P, Benussi S, Kotecha D, Ahlsson A, Atar D, Casadei B, et al. 2016 ESC Guidelines for the management of atrial fibrillation developed in collaboration with EACTS. Eur Heart J. 07 de 2016;37(38):2893–962.

21. World Organization of National Colleges, Academies, and Academic Associations of General Practitioners/Family Physicians, editor. ICPC-2: international classification of primary care. 2nd ed. Oxford ; New York: Oxford University Press; 1998. 190 p. (Oxford medical publications).

22. Ruff CT, Bhatt DL, Steg PG, Gersh BJ, Alberts MJ, Hoffman EB, et al. Long-term cardiovascular outcomes in patients with atrial fibrillation and atherothrombosis in the REACH Registry. Int J Cardiol. 1 january 2014;170(3):413–8.

23. Steffel J, Verhamme P, Potpara TS, Albaladejo P, Antz M, Desteghe L, et al. The 2018 European Heart Rhythm Association Practical Guide on the use of non-vitamin K antagonist oral anticoagulants in patients with atrial fibrillation. Eur Heart J. 21 april 2018;39(16):1330–93.

For peer review only

For peer review only - http://bmjopen.bmj.com/site/about/guidelines.xhtml

428 **TABLES**

429 **Table 1. Characterization of the study population, n= 63,526**

**Gender,** *n (%)*	
Female	33,437 (52.6%)
Male	30,089 (47.4%)
**Age (years),** $\bar{x} \pm s, min, máx$	76.5 ± 10.6, 18, 107
**Age groups,** *n(%)*	
<40 years	286 (0.5)
40-49 years	812 (1.3)
50-59 years	3286 (5.2)
60-69 years	10,089 (15.9)
70-79 years	20,602 (32.4)
≥ 80 years	28,451 (44.8)
**Professional situation,** *n(%)*	
Active	13,553 (21.3)
Not Active	4957 (7.8)
Student	54 (0.1)
Retired	44,685 (70.3)
Unknown	277 (0.5)
**Body Mass Index,** *n(%)*	9448 (14.9%)
**Body Mass Index#,** *Med [Q_1; Q_3]*	29.1 [26.2; 32.6]
**Body Mass Index Categories,** *n(%)*	
**Underweight**	29 (0.3)
**Normal**	1272 (13.5)
**Overweight**	4126 (43.7)
**Obese Class I**	2725 (28.8)
**Obese Class II**	1297 (13.7)
**Glomerular Filtration Rate*,** *n(%)*	
**Value available**	52,207 (82.2%)
**Missing record**	11,319 (17.8%)
**Stage of Chronic Kidney Disease**	
**1**	11,044 (21.2%)
**2**	19,693 (37.7%)
**3**	19,307 (37.0%)
**4**	2110 (4.0%)
**5**	53 (0.1%)
**Cardiovascular risk,** *n(%)*	
**Value available**	51,846 (81.6%)
**Missing record**	11,680 (18.4%)
**Classification of Cardiovascular Risk**	
**Low**	659 (1.3%)
**Moderate**	18,765 (36.2%)
**High**	2347 (4.5%)
**Very high**	30,075 (58.0%)
**CHA_2DS_2-VASc score,** *n(%)*	

For peer review only - http://bmjopen.bmj.com/site/about/guidelines.xhtml

For peer review only

**0 points**	1658 (2.6%)
**1-3 points**	25,843 (40.7%)
**4-5 points**	27,540 (43.3%)
**≥ 6 points**	8485 (13.4%)

#kg/m^2.

*ml/min/1.73 m^2.

For peer review only

Table 2. Prevalence (%) of atrial fibrillation in the northern region of Portugal between 2016 and 2018.

		< 40 years	40-49 years	50-59 years	60-69 years	70-79 years	≥ 80 years	Over 40 years
**2016**	AF patients (n)	200	546	2184	7061	15089	21576	46456
	Population# (n)	1064388	593200	502021	438432	286658	204079	2024390
	Prevalence (%)	**0.019**	**0.092**	**0.435**	**1.611**	**5.264**	**10.572**	**2.295**
**2017**	AF patients (n)	239	678	2647	8442	17665	24746	54178
	Population$ (n)	952914	506129	527554	433722	258239	218169	1943813
	Prevalence (%)	**0.025**	**0.134**	**0.502**	**1.946**	**6.841**	**11.343**	**2.787**
**2018**	AF patients (n)	286	812	3286	10089	20602	28451	63240
	Population£ (n)	957693	588852	530424	432439	307823	218061	2077599
	Prevalence (%)	**0.030**	**0.138**	**0.620**	**2.333**	**6.693**	**13.047**	**3.044**

#Population enrolled in primary health care in northern Portugal in 2016. $Population enrolled in primary health care in northern Portugal in 2017. £Population enrolled in primary health care in northern Portugal in 2018.

For peer review only

For peer review only - http://bmjopen.bmj.com/site/about/guidelines.xhtml

Table 3. Medicated and non-medicated patients with and without a recommendation for anticoagulation.

Recommendation[#]	Medicated, n (%)		Total, n
	Yes	No	
**Yes**	44,326 (72.9)	16,512 (27.1)	60,838
**No**	1100 (40.9)	1588 (59.1)	2688
**Total**	45,426 (71.5)	18,100 (28.5)	63,526

[#]Oral anticoagulation was considered in a male with a CHA_2DS_2-VASc score ≥ 1 point and in a female with a score ≥ 2 points, according to 2016 European Society of Cardiology Guidelines for the management of atrial fibrillation developed in collaboration with European Association of Cardio-Thoracic Surgery.

For peer review only - http://bmjopen.bmj.com/site/about/guidelines.xhtml

Table 4. Distribution of patients for CHA_2DS_2-VASc score value, gender, and respective drugs.

CHA_2DS_2-VASc score	Drugs, *n (%)*					
	**rivaroxaban**	**apixaban**	**dabigatran**	**edoxaban**	**warfarin**	**acenocoumarol**
Male						
**0**	248 (37.1)	134 (20.1)	88 (13.2)	30 (4.5)	127 (19.0)	41 (6.1)
**1**	623 (33.4)	344 (18.4)	276 (14.8)	70 (3.7)	426 (22.8)	129 (6.9)
**2**	1125 (27.3)	801 (19.5)	590 (14.3)	132 (3.2)	1099 (26.7)	369 (9.0)
**3**	1531 (24.4)	1210 (19.3)	823 (13.1)	178 (2.8)	1955 (31.1)	582 (9.3)
**4**	1037 (22.1)	877 (18.7)	547 (11.6)	111 (2.4)	1636 (34.8)	491 (10.4)
**5**	471 (19.7)	486 (20.3)	286 (11.9)	58 (2.4)	813 (33.9)	282 (11.8)
**6**	183 (18.0)	187 (18.4)	102 (10.1)	30 (3.0)	371 (36.6)	141 (13.9)
**7**	61 (19.1)	58 (18.1)	38 (11.9)	7 (2.2)	116 (36.3)	40 (12.5)
**8**	12 (22.6)	8 (15.1)	2 (3.8)	3 (5.7)	20 (37.7)	8 (15.1)
**Subtotal**	5291 (24.7)	4105 (19.2)	2752 (12.9)	619 (2.9)	6563 (30.6)	2083 (9.7)
Female						
**1**	118 (27.3)	88 (20.4)	49 (11.3)	17 (3.9)	125 (28.9)	35 (8.1)
**2**	418 (27.0)	345 (22.3)	189 (12.2)	52 (3.4)	432 (27.9)	110 (7.1)
**3**	951 (24.7)	781 (20.3)	515 (13.4)	152 (3.9)	1130 (29.3)	327 (8.5)
**4**	1708 (23.2)	1565 (21.3)	979 (13.3)	208 (2.8)	2195 (29.9)	694 (9.4)
**5**	1287 (21.3)	1281 (21.2)	694 (11.5)	152 (2.5)	2035 (33.6)	603 (10.0)
**6**	627 (20.8)	623 (20.7)	330 (11.0)	86 (2.9)	1024 (34.0)	322 (10.7)
**7**	232 (18.5)	312 (24.9)	150 (12.0)	28 (2.2)	398 (31.7)	135 (10.8)
**8**	85 (18.8)	121 (26.8)	43 (9.5)	8 (1.8)	148 (32.7)	47 (10.4)
**9**	11 (18.6)	14 (23.7)	6 (10.2)	2 (3.4)	23 (39.0)	3 (5.1)
**Subtotal**	5437 (22.6)	5130 (21.4)	2955 (12.3)	705 (2.9)	7510 (31.3)	2276 (9.5)
**Total**	23.6%	20.3%	12.6%	2.9%	31%	9.6%

For peer review only

Table 5. Distribution of patients for Chronic Kidney Disease' (CKD) stages by respective drugs

**Stages CKD**	**Drugs,** ***n (%)***					
	**rivaroxaban**	**apixaban**	**dabigatran**	**edoxaban**	**warfarin**	**acenocoumarol**
**1**	2164 (27.5)	1531 (19.4)	1081 (13.7)	279 (3.5)	2133 (27.1)	691 (8.8)
**2**	3673 (24.7)	2944 (19.8	1980 (13.3)	434 (2.9)	4428 (29.8)	1386 (9.3)
**3**	3184 (22.3)	3139 (22.0)	1649 (11.6)	394 (2.8)	4504 (31.6)	1393 (9.8)
**4**	202 (14.8)	338 (24.7)	86 (6.3)	28 (2.0)	557 (40.7)	157 (11.5)
**5**	4 (11.1)	11 (30.6)	1 (2.8)	1 (2.8)	17 (47.2)	2 (5.6)
**Total**	9227 (24)	7963 (20.7)	4797 (12.5)	1136 (3.0)	11639 (30.3)	3629 (9.5)

For peer review only

For peer review only - http://bmjopen.bmj.com/site/about/guidelines.xhtml

Table 6. Comorbidities by the International Classification of Primary Care – 2nd Edition.

Comorbidities	n (%)
chronic alcohol abuse (P15)	2392 (3.8)
tobacco abuse (P17)	2880 (4.5)
lipid disorder (T93)	33,095 (52.1)
atherosclerosis/PVD (K92)	4617 (7.3)
diabetes insulin dependente (T89 + T90)	1551 (2.4)
diabetes non-insulin dependente (T90)	16,553 (26.1)
coronary heart disease ischaemic heart disease w. angina (K74)	3370 (5.3)
ischaemic heart disease w/o angina (K76)	3185 (5.0)
acute myocardial infarction (K75)	2450 (3.9)
hypertension complicated (K87)	17,598 (27.7)
hypertension uncomplicated (K86)	31,466 (49.5)
heart failure (K77)	17,530 (27.6)

For peer review only

For peer review only - http://bmjopen.bmj.com/site/about/guidelines.xhtml

STROBE Statement—Checklist of items that should be included in reports of ***cohort studies***

	Item No	Recommendation	Page No
**Title and abstract**	1	(*a*) Indicate the study's design with a commonly used term in the title or the abstract	2
		(*b*) Provide in the abstract an informative and balanced summary of what was done and what was found	2
**Introduction**			
Background/rationale	2	Explain the scientific background and rationale for the investigation being reported	4
Objectives	3	State specific objectives, including any prespecified hypotheses	4
**Methods**			
Study design	4	Present key elements of study design early in the paper	5
Setting	5	Describe the setting, locations, and relevant dates, including periods of recruitment, exposure, follow-up, and data collection	5
Participants	6	(*a*) Give the eligibility criteria, and the sources and methods of selection of participants. Describe methods of follow-up	5
		(*b*) For matched studies, give matching criteria and number of exposed and unexposed	
Variables	7	Clearly define all outcomes, exposures, predictors, potential confounders, and effect modifiers. Give diagnostic criteria, if applicable	5
Data sources/ measurement	8*	For each variable of interest, give sources of data and details of methods of assessment (measurement). Describe comparability of assessment methods if there is more than one group	5
Bias	9	Describe any efforts to address potential sources of bias	NA
Study size	10	Explain how the study size was arrived at	5
Quantitative variables	11	Explain how quantitative variables were handled in the analyses. If applicable, describe which groupings were chosen and why	5
Statistical methods	12	(*a*) Describe all statistical methods, including those used to control for confounding	5
		(*b*) Describe any methods used to examine subgroups and interactions	
		(*c*) Explain how missing data were addressed	
		(*d*) If applicable, explain how loss to follow-up was addressed	
		(*e*) Describe any sensitivity analyses	
**Results**			
Participants	13*	(a) Report numbers of individuals at each stage of study—eg numbers potentially eligible, examined for eligibility, confirmed eligible, included in the study, completing follow-up, and analysed	6
		(b) Give reasons for non-participation at each stage	
		(c) Consider use of a flow diagram	
Descriptive data	14*	(a) Give characteristics of study participants (eg demographic, clinical, social) and information on exposures and potential confounders	6, 17
		(b) Indicate number of participants with missing data for each variable of interest	
		(c) Summarise follow-up time (eg, average and total amount)	
Outcome data	15*	Report numbers of outcome events or summary measures over time	6, 17

For peer review only

For peer review only - http://bmjopen.bmj.com/site/about/guidelines.xhtml

Main results	16	(*a*) Give unadjusted estimates and, if applicable, confounder-adjusted estimates and their precision (eg, 95% confidence interval). Make clear which confounders were adjusted for and why they were included<br>(*b*) Report category boundaries when continuous variables were categorized<br>(*c*) If relevant, consider translating estimates of relative risk into absolute risk for a meaningful time period	6, 20, 21
Other analyses	17	Report other analyses done—eg analyses of subgroups and interactions, and sensitivity analyses	6, 18, 19, 20
**Discussion**			
Key results	18	Summarise key results with reference to study objectives	7
Limitations	19	Discuss limitations of the study, taking into account sources of potential bias or imprecision. Discuss both direction and magnitude of any potential bias	3
Interpretation	20	Give a cautious overall interpretation of results considering objectives, limitations, multiplicity of analyses, results from similar studies, and other relevant evidence	7-8
Generalisability	21	Discuss the generalisability (external validity) of the study results	7-8
**Other information**			
Funding	22	Give the source of funding and the role of the funders for the present study and, if applicable, for the original study on which the present article is based	9

*Give information separately for exposed and unexposed groups.

Note: An Explanation and Elaboration article discusses each checklist item and gives methodological background and published examples of transparent reporting. The STROBE checklist is best used in conjunction with this article (freely available on the Web sites of PLoS Medicine at http://www.plosmedicine.org/, Annals of Internal Medicine at http://www.annals.org/, and Epidemiology at http://www.epidem.com/). Information on the STROBE Initiative is available at http://www.strobe-statement.org.

For peer review only

BMJ Open

AF-React study – Atrial fibrillation management strategies in clinical practice: retrospective longitudinal study from real-world data in Northern Portugal

Journal:	*BMJ Open*
Manuscript ID	bmjopen-2020-040404.R2
Article Type:	Original research
Date Submitted by the Author:	17-Dec-2020
Complete List of Authors:	Silva Pinto, Susana; University of Porto Faculty of Medicine, Family Medicine Unit, Department of Community Medicine, Information and Health Decision Sciences (MEDCIDS); Center for Health Technology and Services Research<br>Teixeira, Andreia; University of Porto Faculty of Medicine, Department of Community Medicine, Information and Health Decision Sciences (MEDCIDS); Center for Health Technology and Services Research<br>Henriques, Teresa; University of Porto Faculty of Medicine, Department of Community Medicine, Information and Health Decision Sciences (MEDCIDS); Center for Health Technology and Services Research<br>Monteiro, Hugo; Research and Planning Department, Regional Health Administration of Northern, Min. of Health Portugal<br>Martins, Carlos; University of Porto Faculty of Medicine, Family Medicine Unit, Department of Community Medicine, Information and Health Decision Sciences (MEDCIDS); Center for Health Technology and Services Research
**Primary Subject Heading**:	Cardiovascular medicine
Secondary Subject Heading:	Epidemiology
Keywords:	PRIMARY CARE, STROKE MEDICINE, Adult cardiology < CARDIOLOGY

SCHOLARONE™
Manuscripts

I, the Submitting Author has the right to grant and does grant on behalf of all authors of the Work (as defined in the below author licence), an exclusive licence and/or a non-exclusive licence for contributions from authors who are: i) UK Crown employees; ii) where BMJ has agreed a CC-BY licence shall apply, and/or iii) in accordance with the terms applicable for US Federal Government officers or employees acting as part of their official duties; on a worldwide, perpetual, irrevocable, royalty-free basis to BMJ Publishing Group Ltd ("BMJ") its licensees and where the relevant Journal is co-owned by BMJ to the co-owners of the Journal, to publish the Work in this journal and any other BMJ products and to exploit all rights, as set out in our licence.

The Submitting Author accepts and understands that any supply made under these terms is made by BMJ to the Submitting Author unless you are acting as an employee on behalf of your employer or a postgraduate student of an affiliated institution which is paying any applicable article publishing charge ("APC") for Open Access articles. Where the Submitting Author wishes to make the Work available on an Open Access basis (and intends to pay the relevant APC), the terms of reuse of such Open Access shall be governed by a Creative Commons licence – details of these licences and which Creative Commons licence will apply to this Work are set out in our licence referred to above.

Other than as permitted in any relevant BMJ Author's Self Archiving Policies, I confirm this Work has not been accepted for publication elsewhere, is not being considered for publication elsewhere and does not duplicate material already published. I confirm all authors consent to publication of this Work and authorise the granting of this licence.

For peer review only

TITLE PAGE

AF-React study – Atrial fibrillation management strategies in clinical practice: retrospective longitudinal study from real-world data in Northern Portugal

Pinto, Susana Silva;[1, 2,3] Email: susyapinto@gmail.com
[1]ACeS Santo Tirso/Trofa, Regional Health Administration of Northern, Min. of Health Portugal.
[2]Department of Community Medicine, Information and Health Decision Sciences (MEDCIDS), Faculty of Medicine, University of Porto, Porto, Portugal.
[3]Centre for Health Technology and Services Research (CINTESIS), University of Porto, Porto, Portugal.

Teixeira, Andreia;[2, 3, 4] Email: andreiasofiat@hotmail.com
[2]Department of Community Medicine, Information and Health Decision Sciences (MEDCIDS), Faculty of Medicine, University of Porto, Porto, Portugal.
[3]Centre for Health Technology and Services Research (CINTESIS), University of Porto, Porto, Portugal.
[4]ARC4DigiT, Polytechnic Institute of Viana do Castelo, Portugal.

Henriques, Teresa S.;[2, 3] Email: teresasarhen@gmail.com
[2]Department of Community Medicine, Information and Health Decision Sciences (MEDCIDS), Faculty of Medicine, University of Porto, Porto, Portugal.
[3]Centre for Health Technology and Services Research (CINTESIS), University of Porto, Porto, Portugal.

Monteiro, Hugo;[5] Email: hfmonteiro@arsnorte.min-saude.pt
[5]Research and Planning Department, Regional Health Administration of Northern, Min. of Health Portugal.

Martins, Carlos.[2, 3] Email: carlosmartins20@gmail.com
[2]Department of Community Medicine, Information and Health Decision Sciences (MEDCIDS), Faculty of Medicine, University of Porto, Porto, Portugal.
[3]Centre for Health Technology and Services Research (CINTESIS), University of Porto, Porto, Portugal.

WORD COUNT

3531

Corresponding Author
Susana Silva Pinto
susyapinto@gmail.com
Rua Dr. Plácido da Costa, s/n 4200-450, Porto, Portugal

For peer review only

ABSTRACT

Objectives
To determine the prevalence of AF and to assess how these patients are being cared for: What anticoagulants are being prescribed and are they being prescribed as recommended?

Design
Retrospective longitudinal study.

Setting
This study was conducted in the Regional Health Administration of Northern Portugal.

Participants
This study used a database that included 63,526 patients with code K78 of the International Classification of Primary Care between January 2016 and December 2018.

Results
The prevalence of AF among adults over 40 years in the northern region of Portugal was 2.3% in 2016, 2.8% in 2017, and 3% in 2018. From a total of 63,526 patients, 95.8% had an indication to receive anticoagulation therapy. Of these, 44,326 (72.9%) are being treated with anticoagulants: 17,936 (40.5%) were prescribed vitamin K antagonists and 26,390 (59.5%) were prescribed non-vitamin K antagonist anticoagulants. On the other hand, 2688 patients of the total (4.2%) had no indication to receive anticoagulation therapy. Of these 2688 patients, 1100 (40.9%) were receiving anticoagulants.

Conclusions
The prevalence of AF is 3%. Here, we report evidence of both undertreatment and overtreatment. Albeit having an indication, a considerable proportion of patients (27.1%) are not anticoagulated, and among AF patients without an indication to receive anticoagulation therapy, a considerable proportion (40.9%) are receiving anticoagulants. The AF-React study brings extremely relevant conclusions to Portugal and follows real-world studies in patients with AF in Europe, presenting some data not yet studied.

Keywords
atrial fibrillation, anticoagulants, prevention, stroke, primary health care.

Strengths and limitations of this study
- The main limitation of this retrospective study is that the AF assessment is based on data coded in the clinical process in Primary Health Care.
- There might be more patients with AF in the northern region of Portugal, but they are not coded at Primary Health Care and, therefore, were not included in this study.
- The lack of ICPC-2 coding for problems such as mechanical prosthesis valve and rheumatic moderate or severe mitral stenosis might be another limitation.
- There might be a small percentage of patients well-medicated with vitamin K antagonists because they are in one of those situations.

  Few of the 63,526 patients will be diagnosed with atrial flutter; however, for patients with atrial flutter, anticoagulant therapy is recommended according to the same risk profile used for AF.

For peer review only

BACKGROUND

Atrial fibrillation (AF) is the most common type of sustained cardiac arrhythmia.(1) The clinical relevance of AF is associated with the loss of effective atrial contractility, resulting in deficient emptying of the left atrial appendage. This process increases the risk of thrombus formation and thromboembolic events.(1)

Due to the ageing of the population, the number of people affected by AF worldwide is projected to exceed 12 million by 2050.(2) In Portugal in 2010, the FAMA study(3) verified an overall prevalence of AF of 2.5% in a population sample older than 40 years, with a significant increase after the age of 70 years.(3)

In Portugal, despite a considerable reduction in ischemic stroke mortality below 70 years (39% reduction) in 2015 compared to 2011, stroke remains the main cause of mortality related to vascular diseases.(4) Patients with AF have a five-fold increased risk of stroke.(5) This arrhythmia is currently responsible for about 15% of stroke cases.(3) Stroke is a major complication associated with AF, which contributes to the morbidity and mortality associated with the disease.(6)

Previous studies have reported that oral anticoagulation with vitamin K antagonists (VKA), such as warfarin, reduces stroke and mortality in AF patients.(7) The non-vitamin K antagonist oral anticoagulants (NOAC) – dabigatran, rivaroxaban, apixaban, edoxaban – have been shown to be superior to warfarin in the prevention of thromboembolic events in patients with non-valvular AF, providing increased safety and an overall reduction in the number of cases of bleeding events(8–11). The cost and morbidity associated with stroke and intracranial haemorrhage are high, so the lower prevalence of these events with NOACs, compared to warfarin, is one factor that might help this drug to become cost-effective relative to warfarin.(12) Furthermore, according to the Portuguese Directorate-General of Health, the reduction in ischemic stroke mortality below 70 years was due to the introduction of NOAC into clinical practice as an antithrombotic therapy for AF. (4)

In a cross-sectional study of patients with AF carried out in eight Family Health Units of Northern Portugal in 2015 (the FATA study),(13) only 56.8% of patients with AF were prescribed adequate oral anticoagulation according to the recommendations of the European Society of Cardiology (ESC) Guidelines in 2010. In the SAFIRA study (2018),(14) 56.3% of patients with AF were not anticoagulated.

There are no randomised controlled trials (RCT) directly comparing NOACs to each other (or to placebo). All RCTs compared a NOAC to warfarin. For example, the RE-LY,(8) ROCKET-AF,(9) ARISTOLETE,(10) and ENGAGE AF-TIMI 48(11) studies included 18,113, 14,264, 18,201, and 21,105 patients with AF, respectively. In fact, these trials were crucial for the efficacy and safety evaluation of these drugs and their approval. However, real-world studies might contribute additional relevant evidence because they include epidemiological data and drug prescribing patterns for a larger number of patients in the context of daily clinical practice. In Portugal, there are no real-world studies with a large database of oral anticoagulation in patients with AF.

In Europe, there are some studies with a large database. A study involving databases from 6 European countries concluded that, overall, apixaban and rivaroxaban increased their use during the study period while dabigatran decreased and there was variability in patient characteristics such as comorbidities, potentially interacting drugs, and dose adjustment.(15) Komen et al. (2017) investigated the influence of patient characteristics such as age, stroke, and bleeding risks on decisions for antithrombotic treatment in patients with AF and concluded that apixaban was favoured for elderly and high-risk patients, whereas dabigatran was used in lower-risk patients.(16) Mueller et al. (2019) compared the clinical effectiveness and safety of NOACs in patients with AF in routine clinical practice. All NOACs were similarly effective at preventing strokes and systemic embolisms, while patients being treated with rivaroxaban exhibited the highest bleeding risks.(17)

For peer review only

The AF-React study aims to determine the prevalence of AF in the northern region of Portugal and to assess how patients with AF are being cared for: What anticoagulants are being prescribed and are they being prescribed as recommended?

For peer review only

METHODS

The Portuguese National Health Service is a universal health coverage system and is administratively divided into five regions: North, Centre, Lisbon and Tagus Valley, Alentejo, and Algarve. The entirety of the mainland Portuguese population is enrolled in one of these administrative health regions. Each regional health administration is responsible for providing primary and secondary healthcare to the population living in its geographic area. This retrospective longitudinal study was conducted using data from the Regional Health Administration database of Northern Portugal.

The Department of Studies and Planning of the Regional Health Administration of Northern Portugal built a database containing the following data for the years 2016, 2017, and 2018: population enrolled in the Regional Health Administration of Northern Portugal, number of patients diagnosed with other ICPC-2 coded health problems,(18) and last record available of body mass index, cardiovascular risk score, and glomerular filtration rate. In fact, this is one-off data (once a year) for all variables except the anticoagulants prescription history, for which we had longitudinal information with the date of each prescription.

To identify AF patients, the International Classification of Primary Care (ICPC-2) code for AF, K78, was used.(18) Therefore, we included all adults (age $\geq$ 18 years) with the code K78 between January and December of the years 2016, 2017, and 2018. For each year, either previous AF diagnosed patients or new AF diagnosed patients were included, so the database comprises both incident and prevalent patients.

The prevalence of AF was calculated for the population enrolled in the Regional Health Administration of Northern Portugal in 2016, 2017, and 2018.

The CV risk (cardiovascular risk) was defined in four stages based on SCORE (Systematic Coronary Risk Evaluation), as low, moderate, high, and very high, if less than 1%, between 1% and 5%, between 5% and 10%, and greater than or equal to 10%, respectively, for patients between 40 and 65 years old.

The stages of CKD were defined based on the glomerular filtration rate (GFR) calculated by the equation of *Cockcroft-Gault*: stage 1, GFR $\geq$ 90 ml/min; stage 2, GFR]90-60] ml/min; stage 3, GFR]60-30] ml/min; stage 4, GFR [30-15] ml/min; and stage 5, GFR < 15ml/min. The high and very high stages of CV risk were based on the codification of health problems by ICPC-2 and the patient's GFR registered in the clinical process.

The body mass index was defined in six categories: <18,5 kg/m^2 – underweight; [18-25[kg/m^2 – normal; [25-30[kg/m^2 – overweight; [30-35[kg/m^2 – obese class I; [35-40[kg/m^2 – obese class II; and $\geq$ 40 kg/m^2 kg/m^2 – obese class III.

The CHA$_2$DS$_2$-VASc score was calculated according to the ESC guidelines in 2016 for AF(19), as follows by ICPC-2 coding (18): C-Congestive heart failure (K77): 1 point; H-Hypertension (K86 or K87): 1 point; A$_2$-Age > 75 years or older: 2 points; D-Diabetes mellitus (T89 or T90): 1 point; S$_2$-Stroke (K89, K90, or K91): 2 points; V-Vascular disease (K75 or K92): 1 point; A-Age 65-74 years: 1 point; and Sc-Sex category (female): 1 point. According to the 2016 ESC guidelines,(19) we considered a CHA$_2$DS$_2$-VASc score $\geq$ 1 point in a male and a CHA$_2$DS$_2$-VASc score $\geq$ 2 points in a female to be an indication of anticoagulation.

The categorical variables were described using absolute and relative frequencies, n (%). The normally distributed continuous variables were described by the mean and respective standard deviation, ± s, and by the minimum (min) and maximum (max) values. In the case of continuous variables not normally distributed, the data are presented by the median and respective interquartile range, Med [Q_1; Q_3], where Q_1 is the first quartile and Q_3 is the third quartile. The normality was verified by observing the histograms.

For peer review only

184 The database was exported to Microsoft Excel 2016® and the statistical analysis was performed
185 using SPSS Statistics 25.0® and software R. Data were obtained without aggregation at the individual
186 level for general characterisation analysis.

187 The data processing was carried out through the Department of Studies and Planning of the
188 Regional Health Administration of Northern Portugal (Ministry of Health, Portugal). The data were
189 extracted from the server through an anonymised data processing and editing platform and delivered
190 securely (and in accordance with legal regulations and due approval) to the principal investigator.
191 Patients and the public were not involved in the development of the research question.

For peer review only

PATIENT AND PUBLIC INVOLVEMENT

Patients and the public were not involved in the development of the research question.

RESULTS

A total of 63,526 patients were identified as having the diagnosis of atrial fibrillation/flutter (ICPC-2, K78 code) in the northern region of Portugal. The average age was 76.5 ± 10.6 years (range from 18 to 107 years) and 53% were female. Table 1 provides a detailed characterisation of this patient group. Considering the 2,077,599 adults over 40 years enrolled in 2018 in the Regional Health Administration of Northern Portugal, the prevalence of AF was 3.044% (Table 2). In 2017, among the 1,943,813 adults over 40 years enrolled in the Regional Health Administration of Northern Portugal, the prevalence of AF was 2.787%. In 2016, among the 2,024,390 adults over 40 years enrolled in the Regional Health Administration of Northern Portugal, the prevalence of AF was 2.295%.

From a total of 63,526 patients, 60,838 (95.8%) had an indication to receive anticoagulation therapy: male with CHA_2DS_2-VASc score ≥ 1 and female with CHA_2DS_2-VASc score ≥ 2. Among these 60,838 patients, 16,512 (27.1%) were not being treated with anticoagulants, and 79% of those 16,512 had a CHA_2DS_2-VASc score of 3 or higher.

Among the 44,326 being treated with anticoagulants, 17,936 (40.5%) were prescribed VKAs (warfarin and acenocoumarol) and 26,390 (59.5%) with NOACs (dabigatran, rivaroxaban, apixaban, and edoxaban). The most commonly prescribed drug in medicated patients was warfarin (31%), followed by rivaroxaban (23.6%), apixaban (20.3%), dabigatran (12.6%), acenocoumarol (9.6%), and edoxaban (2.9%).

The CHA_2DS_2-VASc score has the following distribution: zero points (2.6%), 1–3 points (40.7%), 4–5 points (43.3%), and ≥ 6 points (13.4%). From the total of 30,089 males, 1658 (5.5%) had a CHA_2DS_2-VASc score of zero, and from a total of 33,437 females, 1030 (3.1%) had a CHA_2DS_2-VASc score of 1. This means that these 2688 (4.2%) patients of the total had no indication to receive anticoagulation therapy. Of these 2688 patients, 1100 were receiving anticoagulants (Table 3).

The distributions of the different drugs by CHA_2DS_2-VASc score and gender are shown in Table 4. For males, rivaroxaban (15.0%) and apixaban (8.1%) were more often prescribed than warfarin (7.7%) in the group with a CHA_2DS_2-VASc score of zero. In particular, apixaban was more prescribed than rivaroxaban in groups with CHA_2DS_2-VASc scores of 5 or 6 (15.2% *vs* 14.7% and 13.4% *vs* 13.2%, respectively). For females, apixaban was more prescribed than rivaroxaban in the groups with CHA_2DS_2-VASc scores of 7, 8, or 9 (17.5% *vs* 13.0%, 19.7.8% *vs* 13.9.8%, and 16.7% *vs* 13.1%, respectively).

The CV risk was available for 6531 (72.7%) of the patients between 40 and 65 years old. The CV risk values, for patients in that age group, were distributed as follows: low – 1.0%, moderate – 40.3%, high – 3.0%, and very high – 46.8%.

The glomerular filtration rate was available for 52,207 patients (82.2%) and followed this distribution: stage 1 (21.2%); stage 2 (37.7%); stage 3 (37.0%); stage 4 (4.0%); and stage 5 (0.1%). The VKAs (warfarin and acenocoumarol) were more prescribed than NOACs (dabigatran, rivaroxaban, apixaban, and edoxaban) in stages 4 and 5. The distribution of patients for CKD stages by respective drugs is represented in Table 5.

Of 63,526 patients with AF, between 2016 and 2018, 17,936 (28.2%) were always prescribed VKA and 21,854 (34.4%) were always prescribed NOAC. In particular, 4133 patients (6.5% of the total) had a single recorded prescription for any anticoagulant: warfarin – 1496 (36.2%), rivaroxaban – 882 (21.34%), apixaban – 815 (19.71%), acenocoumarol – 397 (9.61%), dabigatran – 370 (8.95%), and edoxaban – 173 (4.19%). Furthermore, 3508 (5.5%) were prescribed VKA and switched to NOAC, 1504 (2.4%) were prescribed NOAC and switched to another NOAC, and 305 (0.5%) were prescribed NOAC and switched to VKA. The following patients were always prescribed the same NOAC: rivaroxaban – 8801 (22.18%), apixaban – 7052 (17.78%), dabigatran – 5219 (13.16%), and edoxaban – 782 (1.97%).

For peer review only

The prevalence of each of the comorbidities studied in patients with AF is represented in Table 6. We found that the most prevalent comorbidities associated with AF were hypertension (77.2%), dyslipidemia (52.1%), diabetes (28.5%), and heart failure (27.6%).

For peer review only

DISCUSSION

Summary

In the AF-React study, the prevalence of AF was 3% in 2018. A considerable proportion of patients, with an indication for anticoagulation therapy, are not anticoagulated (27.1%), and among AF patients without indication to receive anticoagulation therapy, a considerable proportion (40.9%) are receiving anticoagulants.

Comparison with existing literature

The prevalence of AF found in this study is similar to that of the FAMA study(3) (3% *vs* 2.5%), and higher than that of the FATA study(13) (3% *vs* 1.3%). The FAMA study(3) used a representative sample of the Portuguese population over 40 years old to study the prevalence of AF in 2010. The AF disease was determined using a 12-lead electrocardiogram. The FATA study(13) is an observational cross-sectional study from 2014 that included all patients aged 30 or above diagnosed with AF, enrolled in one of the eight Family Health Units of a northern region of Portugal. The higher values found in our study, as compared to the FATA study,(13) corroborate our results: The prevalence of AF is increasing with time in the northern region of Portugal. As far as following expectations, this might also be related to the improvement of the coding process of the problems in the clinical process. It was found that the majority of AF patients in this study are elderly, as in the ATRIA study(4) (44.8% were aged 80 years or older *vs* 45% were aged 75 years or older).

The CHA_2DS_2-VASc scores found in our study are similar to those of the FATA study(13): zero points (2.6% *vs* 2.3%), 1–3 points (40.7% *vs* 40.9%), 4–5 points (43.3% *vs* 41.6%), and $\geq$ 6 (13.4% *vs* 14.3%). In our study, 44,326 (72.9%) patients were adequately anticoagulated, which is an improvement relative to the FAMA,(3) FATA,(13) SAFIRA,(14) and REACH (20) studies (38%, 56.8%, 43.7%, and 54%, respectively). These studies were published prior to the 2016 ESC guidelines.(19) The guidelines advise that NOAC should be considered for men with a CHA_2DS_2-VASc score $\geq$ 1 and women with a score $\geq$ 2, which might have contributed to the improvement in anticoagulation in AF patients. It should be noted that, in our study, 79% of patients with AF who are not anticoagulated have a high thrombotic risk (CHA_2DS_2-VASc score $\geq$ 3). This information is of most importance because clinicians should be proactive in the prevention of stroke in patients with AF.

In addition to evidence of anticoagulation undertreatment in AF patients, overtreatment is also found. In our study, 40.9% of AF patients who do not have an indication for anticoagulation are taking anticoagulants. Similar overtreatment rates occur in the REACH study,(20) where 43% of patients with AF and not indicated for anticoagulation were receiving anticoagulants. The FAMA,(3) FATA,(13) and SAFIRA(14) studies did not report overtreatment rates. A possible explanation for this overtreatment rate in our study might be a misuse of the CHA_2DS_2-VASc score. Some of these patients might also be prescribed an anticoagulation agent for other clinical reasons. Hess, P.L. et al. (2014) identified a broad range of barriers to oral anticoagulation, including knowledge gaps about stroke risk and the relative risks and benefits of anticoagulant therapies; lack of awareness regarding the potential use of NOAC agents for VKA-unsuitable patients; lack of recognition of expanded eligibility for oral anticoagulation; lack of availability of reversal agents and the difficulty of anticoagulant effect monitoring for the NOACs; concerns with the bleeding risk of anticoagulant therapy, especially with the NOACs and particularly in the setting of dual antiplatelet therapy; suboptimal time in therapeutic range for VKA; and costs and insurance coverage.(21)

Although there has been a positive evolution regarding the number of patients with anticoagulated AF, warfarin remains the most prescribed anticoagulant. As noted before, this is not concordant with the 2016 ESC guidelines.(19) When analysing the distribution of drugs by CHA_2DS_2-VASc score and gender, it should be noted that in men who are not indicated to be anticoagulated (CHA_2DS_2-VASc score of zero), the most prescribed drug is rivaroxaban, which might result from another

For peer review only

therapeutic indication, such as deep venous thrombosis. On the other hand, acenocoumarol is more prescribed than dabigatran both in men with CHA_2DS_2-VASc scores of 7 and 8 and in women with a CHA_2DS_2-VASc score of 8. These results might be explained by the fact that clinicians prefer drugs with which they have more experience, such as VKA in patients with a very high thrombotic risk. However, this does not follow the most current guidelines.

The SAFIRA(14) study showed a very high prevalence of CV risk factors in patients with AF. In Portugal, SCORE is recommended for use in calculating CV risk in those between 40 and 65 years old.(22,23) However, we found no study that calculated CV risk by SCORE. The CV risk of patients aged 40–65 years was considered, with most patients having a very high CV risk. In fact, according to the 2016 ESC guidelines,(19) most patients with AF have many other comorbidities, so we consider this to be a reason for having a much larger number of very high risk than high risk patients in our study.

The SAFIRA(14) study found that 24.7% of the patients were incorrectly medicated (inadequate dose, inadequate number of doses, or creatinine clearance incompatible with the use of NOAC), but it did not discriminate patients by stages of CKD. The AF-React study showed that NOACs are being prescribed in stage 5 of CKD, which was contraindicated by the guidelines in force at the date of prescription (between 2016 to 2018).(18,24) In stage 4 of CKD, the 2016 ESC Guidelines(19) do not recommend the use of NOACs; the EHRA practical guide(24) recommends dose adjustment for apixaban, rivaroxaban, and edoxaban and does not recommend the use of dabigatran. This data opens the door to a more detailed analysis in the future about the prescriptions in stage 4.

Limitations

The main limitation of this retrospective study is that the AF assessment is based on data coded in the clinical process in Primary Health Care. There might be more patients with AF in the northern region of Portugal, but they are not coded at Primary Health Care and, therefore, were not included in this study. Hence, strict coding by all family doctors is important. Another limitation might be the lack of ICPC-2 coding for problems such as mechanical prosthesis valve and rheumatic moderate or severe mitral stenosis(19). There might be a small percentage of patients who are well medicated with vitamin K antagonists because they are in one of those situations.

The code K78, ICPC-2 included the diagnosis of AF and atrial flutter. Few of the 63,526 patients will be diagnosed with atrial flutter, so this is a coding limitation of ICPC-2. However, this limitation does not invalidate the results of our study because, for patients with atrial flutter, anticoagulant therapy is recommended according to the same risk profile used for AF.(25)

Implications for research and practice

The AF-React study made it possible to determine the prevalence of AF and to evaluate how patients with AF are treated in northern Portugal. Thus, the AF-React study brings extremely relevant conclusions to Portugal and follows real-world studies in patients with AF in Europe, presenting some data not yet studied: distribution of patients for CHA_2DS_2-VASc score value, gender and respective drugs, classification of CV risk and distribution of patients for chronic kidney disease stages by respective drugs. For the future, it would be necessary for health authorities to allow continued access to these data sets, including mortality data, to draw conclusions (based on the real world) about the impact of the new treatments on patient-oriented outcomes.

For peer review only

FUNDING

This article was supported by National Funds through FCT - Fundação para a Ciência e a Tecnologia I.P., within CINTESIS, R&D Unit (reference UIDB/IC/4255/2020). The authors have received financial support of €4,400 EUR from the #H4A Primary Healthcare Research Network Scholars Program research support.

For peer review only

ETHICAL APPROVAL

The study protocol was approved by the Health Ethics Committee of the Regional Health Administration of Northern Portugal.

For peer review only

COMPETING INTERESTS

The authors declared no potential conflicts of interest with respect to the research, authorship, and/or publication of this article.

For peer review only

ACKNOWLEDGEMENTS

A special thanks to all primary healthcare professionals who in their daily work in the Portuguese Health System fulfil the clinical records and allow researchers like us to obtain evidence that will help us to improve the health of the Portuguese.

For peer review only

AUTHOR CONTRIBUTIONS

SSP and CM contributed to the conception of the work; all authors contributed to the design of the study. HM contributed to the data acquisition process; SPP, AT, and TH contributed to the analysis and interpretation of the data. SSP drafted the manuscript, HM, AT, TH, and CM critically revised the manuscript. All authors gave final approval and agreed to be accountable for all aspects of the work, ensuring integrity and accuracy.

For peer review only

DATA SHARING STATEMENT

Data are available upon reasonable request.

For peer review only

REFERENCES

1. Falk RH. Atrial fibrillation. *N Engl J Med* 2001;344(14):1067–78.

2. Shafeeq H, Tran TH. New oral anticoagulants for atrial fibrillation: are they worth the risk? *P T Peer-Rev J Formul Manag* 2014;39(1):54–64.

3. Bonhorst D, Mendes M, Adragão P, et al. Prevalence of atrial fibrillation in the Portuguese population aged 40 and over: the FAMA study. *Rev Port Cardiol.* 2010;29(3):331–50.

4. Ferreira RC, Macedo ME, Pinto F, et al. PROGRAMA NACIONAL PARA AS DOENÇAS CÉREBRO-CARDIOVASCULARES 2017 [Internet]. Direção Geral da Saúde; 2017.

5. da Silva RMFL. Novel oral anticoagulants in non-valvular atrial fibrillation. *Cardiovasc Hematol Agents Med Chem* 2014;12(1):3–8.

6. Alonso A, Norby FL. Predicting Atrial Fibrillation and Its Complications. *Circ J Off J Jpn Circ Soc* 2016;80(5):1061–6.

7. Aguilar MI, Hart R. Oral anticoagulants for preventing stroke in patients with non-valvular atrial fibrillation and no previous history of stroke or transient ischemic attacks. Cochrane Database Syst Rev. 2005;(3):CD001927.

8. Connolly SJ, Ezekowitz MD, Yusuf S, et al. Dabigatran versus warfarin in patients with atrial fibrillation. *N Engl J Med* 2009;361(12):1139–51.

9. Patel MR, Mahaffey KW, Garg J, et al. Rivaroxaban versus warfarin in nonvalvular atrial fibrillation. *N Engl J Med* 2011;365(10):883–91.

10. Granger CB, Alexander JH, McMurray JJV, et al. Apixaban versus warfarin in patients with atrial fibrillation. *N Engl J Med* 2011;365(11):981–92.

11. Giugliano RP, Ruff CT, Braunwald E, et al. Edoxaban versus warfarin in patients with atrial fibrillation. *N Engl J Med* 2013;369(22):2093–104.

12. Matchar DB, Samsa GP, Cohen SJ, et al. Improving the quality of anticoagulation of patients with atrial fibrillation in managed care organizations: results of the managing anticoagulation services trial. *Am J Med* 2002;113(1):42–51.

13. Gomes E, Campos R, Morais R, et al. [FATA Study: prevalence of atrial fibrillation and antithrombotic therapy in primary health care in a northern city of Portugal]. *Acta Med Port* 2015;28(1):35–43.

14. Monteiro P. Estudo Safira: reflexões sobre a prevalência e os padrões de tratamento de fibrilhação auricular e risco cardiovascular em 7500 indivíduos com 65 ou mais anos. *Rev Port Cardiol* 2018;37(4):307–13.

15. Ibáñez L, Sabaté M, Vidal X, et al. Incidence of direct oral anticoagulant use in patients with nonvalvular atrial fibrillation and characteristics of users in 6 European countries (2008–2015): A cross-national drug utilization study. *Br J Clin Pharmacol* 2019;85(11):2524–39.

For peer review only

16. Komen J, Forslund T, Hjemdahl P, et al. Factors associated with antithrombotic treatment decisions for stroke prevention in atrial fibrillation in the Stockholm region after the introduction of NOACs. *Eur J Clin Pharmacol* 2017;73(10):1315–22.

17. Mueller T, Alvarez-Madrazo S, Robertson C, et al. Comparative safety and effectiveness of direct oral anticoagulants in patients with atrial fibrillation in clinical practice in Scotland. *Br J Clin Pharmacol* 2019;85(2):422–31.

18. World Organization of National Colleges, Academies, and Academic Associations of General Practitioners/Family Physicians, editor. ICPC-2: international classification of primary care. 2nd ed. Oxford ; New York: Oxford University Press; 1998. 190 p. (Oxford medical publications).

19. Kirchhof P, Benussi S, Kotecha D, et al. 2016 ESC Guidelines for the management of atrial fibrillation developed in collaboration with EACTS. *Eur Heart J* 2016;37(38):2893–962.

20. Ruff CT, Bhatt DL, Steg PG, et al. Long-term cardiovascular outcomes in patients with atrial fibrillation and atherothrombosis in the REACH Registry. *Int J Cardiol* 2014;170(3):413–8.

21. Hess PL, Mirro MJ, Diener H-C, et al. Addressing barriers to optimal oral anticoagulation use and persistence among patients with atrial fibrillation: Proceedings, Washington, DC, December 3-4, 2012. *Am Heart J* 2014;168(3):239-247.e1.

22. European Association for Cardiovascular Prevention & Rehabilitation, Reiner Z, Catapano AL, De Backer G, et al. ESC/EAS Guidelines for the management of dyslipidaemias: the Task Force for the management of dyslipidaemias of the European Society of Cardiology (ESC) and the European Atherosclerosis Society (EAS). *Eur Heart J* 2011;32(14):1769–818.

23. Authors/Task Force Members: Piepoli MF, Hoes AW, Agewall S, et al. 2016 European Guidelines on cardiovascular disease prevention in clinical practice: The Sixth Joint Task Force of the European Society of Cardiology and Other Societies on Cardiovascular Disease Prevention in Clinical Practice (constituted by representatives of 10 societies and by invited experts) Developed with the special contribution of the European Association for Cardiovascular Prevention & Rehabilitation (EACPR). *Atherosclerosis* 2016;252:207–74.

24. Steffel J, Verhamme P, Potpara TS, et al. The 2018 European Heart Rhythm Association Practical Guide on the use of non-vitamin K antagonist oral anticoagulants in patients with atrial fibrillation. *Eur Heart J* 2018;39(16):1330–93.

25. January CT, Wann LS, Calkins H, et al. 2019 AHA/ACC/HRS Focused Update of the 2014 AHA/ACC/HRS Guideline for the Management of Patients With Atrial Fibrillation: A Report of the American College of Cardiology/American Heart Association Task Force on Clinical Practice Guidelines and the Heart Rhythm Society in Collaboration With the Society of Thoracic Surgeons. *Circulation* [Internet]. 2019.

For peer review only

442 TABLES

443 **Table 1. Characterization of the atrial fibrillation patients diagnosed in the Regional Health**
444 **Administration of Northern Portugal (n= 63,526).**

**Gender,** *n (%)*	
Female	33,437 (52.6%)
Male	30,089 (47.4%)
**Age (years),** $\bar{x} \pm s, min, máx$	76.5 ± 10.6, 18, 107
**Age groups,** *n(%)*	
<40 years	286 (0.5)
40-49 years	812 (1.3)
50-59 years	3286 (5.2)
60-69 years	10,089 (15.9)
70-79 years	20,602 (32.4)
≥ 80 years	28,451 (44.8)
**Professional situation,** *n(%)*	
Active	13,553 (21.3)
Not Active	4957 (7.8)
Student	54 (0.1)
Retired	44,685 (70.3)
Unknown	277 (0.5)
**Body Mass Index,** *n(%)*	9448 (14.9%)
**Body Mass Index**[#]**,** *Med [Q_1; Q_3]*	29.1 [26.2; 32.6]
**Body Mass Index Categories,** *n(%)*	
**Underweight**	29 (0.3)
**Normal**	1272 (13.5)
**Overweight**	4126 (43.7)
**Obese Class I**	2725 (28.8)
**Obese Class II**	1297 (13.7)
**Glomerular Filtration Rate*,** *n(%)*	
**Value available**	52,207 (82.2%)
**Missing record**	11,319 (17.8%)
**Stage of Chronic Kidney Disease**	
**1**	11,044 (21.2%)
**2**	19,693 (37.7%)
**3**	19,307 (37.0%)
**4**	2110 (4.0%)
**5**	53 (0.1%)
**Cardiovascular risk for patients with age between 40-65 years (n =8985),** *n(%)*	
**Value available**	6531 (72.7%)
**Missing record**	2454 (27.3%)

For peer review only

**Classification of Cardiovascular Risk[¥] (n =6531),** *n(%)*	
**Low**	650 (10.0%)
**Moderate**	2631 (40.3%)
**High**	194 (3.0%)
**Very high**	3056 (46.7%)
**CHA_2DS_2-VASc score,** *n(%)*	
**0**	1658 (2.6%)
**1**	3967 (6.2%)
**2**	8065 (12.7%)
**3**	13811 (21.7%)
**4**	16152 (25.4%)
**5**	11388 (17.9%)
**6**	5520 (8.7%)
**7**	2196 (3.5%)
**8**	685 (1.1%)
**9**	84 (0.1%)

[#] The body mass index is defined under six categories: <18,5 kg/m2 – underweight; [18-25[kg/m2 – normal; [25-30[kg/m2 – overweight; [30-35[kg/m2 – obese class I; [35-40[kg/m2 – obese class II; ≥ 40 kg/m2 kg/m2 – obese class III.

* The stages of CKD were defined based on the glomerular filtration rate (GFR) calculated by the equation of *Cockcroft-Gault*: stage 1, GFR ≥ 90 ml/min; stage 2, GFR]90-60] ml/min; stage 3, GFR]60-30] ml/min, stage 4, GFR [30-15] ml/min; and stage 5, GFR < 15ml/min.

¥ The cardiovascular risk was defined under four stages that were based on SCORE (Systematic Coronary Risk Evaluation), as low, moderate, high, and very high, if less than 1%, between 1% and 5%, between 5% and 10%, and greater than or equal to 10%.

For peer review only

Table 2. Prevalence (%) of atrial fibrillation in the northern region of Portugal between 2016 and 2018.

		< 40 years	40-49 years	50-59 years	60-69 years	70-79 years	≥ 80 years	Over 40 years
**2016**	AF patients (n)	200	546	2184	7061	15089	21576	46456
	Population# (n)	1064388	593200	502021	438432	286658	204079	2024390
	Prevalence (%)	**0.019**	**0.092**	**0.435**	**1.611**	**5.264**	**10.572**	**2.295**
**2017**	AF patients (n)	239	678	2647	8442	17665	24746	54178
	Population$ (n)	952914	506129	527554	433722	258239	218169	1943813
	Prevalence (%)	**0.025**	**0.134**	**0.502**	**1.946**	**6.841**	**11.343**	**2.787**
**2018**	AF patients (n)	286	812	3286	10089	20602	28451	63240
	Population£ (n)	957693	588852	530424	432439	307823	218061	2077599
	Prevalence (%)	**0.030**	**0.138**	**0.620**	**2.333**	**6.693**	**13.047**	**3.044**

#Population enrolled in primary health care in northern Portugal based on the age pyramid of Regional Health Administration of Northern Portugal in 2016. $Population enrolled in primary health care in northern Portugal based on the age pyramid of Regional Health Administration of Northern Portugal in 2017. £Population enrolled in primary health care in northern Portugal based on the age pyramid of Regional Health Administration of Northern Portugal in 2018.

For peer review only

Table 3. Medicated and non-medicated patients with and without a recommendation for anticoagulation.

Recommendation[#]	Medicated, n (%)		Total, n
	Yes	No	
**Yes**	44,326 (72.9)	16,512 (27.1)	60,838
**No**	1100 (40.9)	1588 (59.1)	2688
**Total**	45,426 (71.5)	18,100 (28.5)	63,526

[#]Oral anticoagulation was considered in a male with a CHA_2DS_2-VASc score ≥ 1 point and in a female with a score ≥ 2 points, according to 2016 European Society of Cardiology Guidelines for the management of atrial fibrillation developed in collaboration with European Association of Cardio-Thoracic Surgery.

For peer review only

Table 4. Distribution of patients for CHA_2DS_2-VASc score value, gender, and respective drugs.

CHA_2DS_2-VASc score	Drugs, *n (%)*								
	No OAC*	NOAC[#] group					VKA group		
	Total	Total	rivaroxaban	apixaban	dabigatran	edoxaban	Total	warfarin	acenocoumarol
Male									
**0**	990 (59.7)	500 (30.2)	248 (15.0)	134 (8.1)	88 (5.3)	30 (1.8)	168 (10.1)	127 (7.7)	41 (2.5)
**1**	1069 (36.4)	1313 (44.7)	623 (21.2)	344 (11.7)	276 (9.4)	70 (2.4)	555 (18.9)	426 (14.5)	129 (4.4)
**2**	1646 (28.6)	2648 (46.0)	1125 (19.5)	801 (13.9)	590 (10.2)	132 (2.3)	1468 (25.5)	1099 (19.1)	369 (6.4)
**3**	2132 (25.3)	3742 (44.5)	1531 (18.2)	1210 (14.4)	823 (9.8)	178 (2.1)	2537 (30.2)	1955 (23.2)	582 (6.9)
**4**	1549 (24.8)	2572 (41.2)	1037 (16.6)	877 (14.0)	547 (8.8)	111 (1.8)	2127 (34.0)	1636 (26.2)	491 (7.9)
**5**	798 (25.0)	1301 (40.7)	471 (14.7)	486 (15.2)	286 (9.0)	58 (1.8)	1095 (34.3)	813 (25.5)	282 (8.8)
**6**	377 (27.1)	502 (36.1)	183 (13.2)	187 (13.4)	102 (7.3)	30 (2.2)	512 (36.8)	371 (26.7)	141 (10.1)
**7**	96 (23.1)	164 (39.4)	61 (14.7)	58 (13.9)	38 (9.1)	7 (1.7)	156 (37.5)	116 (27.9)	40 (9.6)
**8**	19 (26.4)	25 (34.7)	12 (16.7)	8 (11.1)	2 (2.8)	3 (4.2)	28 (38.9)	20 (27.8)	8 (11.1)
**Subtotal**	8676 (28.8)	12767 (42.4)	5291 (17.6)	4105 (13.6)	2752 (9.1)	619 (2.1)	8646 (28.7)	6563 (21.8)	2083 (6.9)
Female									
**1**	598 (58.1)	272 (26.4)	118 (11.5)	88 (8.5)	49 (4.8)	17 (1.7)	160 (15.5)	125 (12.1)	35 (3.4)
**2**	757 (32.9)	1004 (43.6)	418 (18.2)	345 (15.0)	189 (8.2)	52 (2.3)	542 (23.5)	432 (18.8)	110 (4.8)
**3**	1544 (28.6)	2399 (44.4)	951 (17.6)	781 (14.5)	515 (9.5)	152 (2.8)	1457 (27.0)	1130 (20.9)	327 (6.1)
**4**	2555 (25.8)	4460 (45.0)	1708 (17.2)	1565 (15.8)	979 (9.9)	208 (2.1)	2889 (29.2)	2195 (22.2)	694 (7.0)
**5**	2142 (26.1)	3369 (41.3)	1287 (15.7)	1281 (15.6)	694 (8.5)	152 (1.9)	2638 (32.4)	2035 (24.8)	603 (7.4)
**6**	1117 (27.1)	1666 (40.3)	627 (15.2)	623 (15.1)	330 (8.0)	86 (2.1)	1346 (32.6)	1024 (24.8)	322 (7.8)
**7**	525 (29.5)	722 (40.6)	232 (13.0)	312 (17.5)	150 (8.4)	28 (1.6)	533 (29.9)	398 (22.4)	135 (7.6)
**8**	161 (26.3)	257 (41.9)	85 (13.9)	121 (19.7)	43 (7.0)	8 (1.3)	195 (31.8)	148 (24.1)	47 (7.7)
**9**	25 (29.8)	33 (39.3)	11 (13.1)	14 (16.7)	6 (7.1)	2 (2.4)	26 (31.0)	23 (27.4)	3 (3.6)
**Subtotal**	9424 (28.2)	14227 (42.5)	5437 (16.3)	5130 (15.3)	2955 (8.8)	705 (2.1)	9786 (29.3)	7510 (22.5)	2276 (6.8)
**Total**	18100 (28.5)	26994 (42.5)	10728 (16.9)	9235 (14.5)	5707 (9.0)	1324 (2.1)	18432 (29.0)	14073 (22.2)	4359 (6.9)

*OAC: oral anticoagulation

[#] NOAC: non-vitamin K antagonist oral anticoagulants

[$] VKA: vitamin K antagonists

For peer review only

Table 5. Distribution of patients for Chronic Kidney Disease' (CKD) stages by respective drugs

Stages CKD	Drugs, *n (%)*					
	**rivaroxaban**	**apixaban**	**dabigatran**	**edoxaban**	**warfarin**	**acenocoumarol**
**1**	2164 (27.5)	1531 (19.4)	1081 (13.7)	279 (3.5)	2133 (27.1)	691 (8.8)
**2**	3673 (24.7)	2944 (19.8	1980 (13.3)	434 (2.9)	4428 (29.8)	1386 (9.3)
**3**	3184 (22.3)	3139 (22.0)	1649 (11.6)	394 (2.8)	4504 (31.6)	1393 (9.8)
**4**	202 (14.8)	338 (24.7)	86 (6.3)	28 (2.0)	557 (40.7)	157 (11.5)
**5**	4 (11.1)	11 (30.6)	1 (2.8)	1 (2.8)	17 (47.2)	2 (5.6)
**Total**	9227 (24)	7963 (20.7)	4797 (12.5)	1136 (3.0)	11639 (30.3)	3629 (9.5)

For peer review only

Table 6. Comorbidities by the International Classification of Primary Care – 2nd Edition.

Comorbidities	n (%)
chronic alcohol abuse (P15)	2392 (3.8)
tobacco abuse (P17)	2880 (4.5)
lipid disorder (T93)	33,095 (52.1)
atherosclerosis/PVD (K92)	4617 (7.3)
diabetes insulin dependente (T89 + T90)	1551 (2.4)
diabetes non-insulin dependente (T90)	16,553 (26.1)
coronary heart disease ischaemic heart disease w. angina (K74)	3370 (5.3)
ischaemic heart disease w/o angina (K76)	3185 (5.0)
acute myocardial infarction (K75)	2450 (3.9)
hypertension complicated (K87)	17,598 (27.7)
hypertension uncomplicated (K86)	31,466 (49.5)
heart failure (K77)	17,530 (27.6)

For peer review only

STROBE Statement—Checklist of items that should be included in reports of ***cohort studies***

	Item No	Recommendation	Page No
**Title and abstract**	1	(*a*) Indicate the study's design with a commonly used term in the title or the abstract	2
		(*b*) Provide in the abstract an informative and balanced summary of what was done and what was found	2
**Introduction**			
Background/rationale	2	Explain the scientific background and rationale for the investigation being reported	4
Objectives	3	State specific objectives, including any prespecified hypotheses	4
**Methods**			
Study design	4	Present key elements of study design early in the paper	5
Setting	5	Describe the setting, locations, and relevant dates, including periods of recruitment, exposure, follow-up, and data collection	5
Participants	6	(*a*) Give the eligibility criteria, and the sources and methods of selection of participants. Describe methods of follow-up	5
		(*b*) For matched studies, give matching criteria and number of exposed and unexposed	
Variables	7	Clearly define all outcomes, exposures, predictors, potential confounders, and effect modifiers. Give diagnostic criteria, if applicable	5
Data sources/ measurement	8*	For each variable of interest, give sources of data and details of methods of assessment (measurement). Describe comparability of assessment methods if there is more than one group	5
Bias	9	Describe any efforts to address potential sources of bias	NA
Study size	10	Explain how the study size was arrived at	5
Quantitative variables	11	Explain how quantitative variables were handled in the analyses. If applicable, describe which groupings were chosen and why	5
Statistical methods	12	(*a*) Describe all statistical methods, including those used to control for confounding	5
		(*b*) Describe any methods used to examine subgroups and interactions	
		(*c*) Explain how missing data were addressed	
		(*d*) If applicable, explain how loss to follow-up was addressed	
		(*e*) Describe any sensitivity analyses	
**Results**			
Participants	13*	(a) Report numbers of individuals at each stage of study—eg numbers potentially eligible, examined for eligibility, confirmed eligible, included in the study, completing follow-up, and analysed	6
		(b) Give reasons for non-participation at each stage	
		(c) Consider use of a flow diagram	
Descriptive data	14*	(a) Give characteristics of study participants (eg demographic, clinical, social) and information on exposures and potential confounders	6, 17
		(b) Indicate number of participants with missing data for each variable of interest	
		(c) Summarise follow-up time (eg, average and total amount)	
Outcome data	15*	Report numbers of outcome events or summary measures over time	6, 17

For peer review only

Main results	16	(*a*) Give unadjusted estimates and, if applicable, confounder-adjusted estimates and their precision (eg, 95% confidence interval). Make clear which confounders were adjusted for and why they were included<br>(*b*) Report category boundaries when continuous variables were categorized<br>(*c*) If relevant, consider translating estimates of relative risk into absolute risk for a meaningful time period	6, 20, 21
Other analyses	17	Report other analyses done—eg analyses of subgroups and interactions, and sensitivity analyses	6, 18, 19, 20
**Discussion**			
Key results	18	Summarise key results with reference to study objectives	7
Limitations	19	Discuss limitations of the study, taking into account sources of potential bias or imprecision. Discuss both direction and magnitude of any potential bias	3
Interpretation	20	Give a cautious overall interpretation of results considering objectives, limitations, multiplicity of analyses, results from similar studies, and other relevant evidence	7-8
Generalisability	21	Discuss the generalisability (external validity) of the study results	7-8
**Other information**			
Funding	22	Give the source of funding and the role of the funders for the present study and, if applicable, for the original study on which the present article is based	9

*Give information separately for exposed and unexposed groups.

Note: An Explanation and Elaboration article discusses each checklist item and gives methodological background and published examples of transparent reporting. The STROBE checklist is best used in conjunction with this article (freely available on the Web sites of PLoS Medicine at http://www.plosmedicine.org/, Annals of Internal Medicine at http://www.annals.org/, and Epidemiology at http://www.epidem.com/). Information on the STROBE Initiative is available at http://www.strobe-statement.org.

For peer review only

BMJ Open

AF-React study – Atrial fibrillation management strategies in clinical practice: retrospective longitudinal study from real-world data in Northern Portugal

Journal:	*BMJ Open*
Manuscript ID	bmjopen-2020-040404.R3
Article Type:	Original research
Date Submitted by the Author:	18-Jan-2021
Complete List of Authors:	Silva Pinto, Susana; University of Porto Faculty of Medicine, Family Medicine Unit, Department of Community Medicine, Information and Health Decision Sciences (MEDCIDS); Center for Health Technology and Services Research<br>Teixeira, Andreia; University of Porto Faculty of Medicine, Department of Community Medicine, Information and Health Decision Sciences (MEDCIDS); Center for Health Technology and Services Research<br>Henriques, Teresa; University of Porto Faculty of Medicine, Department of Community Medicine, Information and Health Decision Sciences (MEDCIDS); Center for Health Technology and Services Research<br>Monteiro, Hugo; Research and Planning Department, Regional Health Administration of Northern, Min. of Health Portugal<br>Martins, Carlos; University of Porto Faculty of Medicine, Family Medicine Unit, Department of Community Medicine, Information and Health Decision Sciences (MEDCIDS); Center for Health Technology and Services Research
**Primary Subject Heading**:	Cardiovascular medicine
Secondary Subject Heading:	Epidemiology
Keywords:	PRIMARY CARE, STROKE MEDICINE, Adult cardiology < CARDIOLOGY

SCHOLARONE™
Manuscripts

I, the Submitting Author has the right to grant and does grant on behalf of all authors of the Work (as defined in the below author licence), an exclusive licence and/or a non-exclusive licence for contributions from authors who are: i) UK Crown employees; ii) where BMJ has agreed a CC-BY licence shall apply, and/or iii) in accordance with the terms applicable for US Federal Government officers or employees acting as part of their official duties; on a worldwide, perpetual, irrevocable, royalty-free basis to BMJ Publishing Group Ltd ("BMJ") its licensees and where the relevant Journal is co-owned by BMJ to the co-owners of the Journal, to publish the Work in this journal and any other BMJ products and to exploit all rights, as set out in our licence.

The Submitting Author accepts and understands that any supply made under these terms is made by BMJ to the Submitting Author unless you are acting as an employee on behalf of your employer or a postgraduate student of an affiliated institution which is paying any applicable article publishing charge ("APC") for Open Access articles. Where the Submitting Author wishes to make the Work available on an Open Access basis (and intends to pay the relevant APC), the terms of reuse of such Open Access shall be governed by a Creative Commons licence – details of these licences and which Creative Commons licence will apply to this Work are set out in our licence referred to above.

Other than as permitted in any relevant BMJ Author's Self Archiving Policies, I confirm this Work has not been accepted for publication elsewhere, is not being considered for publication elsewhere and does not duplicate material already published. I confirm all authors consent to publication of this Work and authorise the granting of this licence.

For peer review only

TITLE PAGE

AF-React study – Atrial fibrillation management strategies in clinical practice: retrospective longitudinal study from real-world data in Northern Portugal

Pinto, Susana Silva;[1, 2,3] Email: susyapinto@gmail.com
[1]ACeS Santo Tirso/Trofa, Regional Health Administration of Northern, Min. of Health Portugal.
[2]Department of Community Medicine, Information and Health Decision Sciences (MEDCIDS), Faculty of Medicine, University of Porto, Porto, Portugal.
[3]Centre for Health Technology and Services Research (CINTESIS), University of Porto, Porto, Portugal.

Teixeira, Andreia;[2, 3, 4] Email: andreiasofiat@hotmail.com
[2]Department of Community Medicine, Information and Health Decision Sciences (MEDCIDS), Faculty of Medicine, University of Porto, Porto, Portugal.
[3]Centre for Health Technology and Services Research (CINTESIS), University of Porto, Porto, Portugal.
[4]Instituto Politécnico de Viana do Castelo, Viana do Castelo, Portugal.

Henriques, Teresa S.;[2, 3] Email: teresasarhen@gmail.com
[2]Department of Community Medicine, Information and Health Decision Sciences (MEDCIDS), Faculty of Medicine, University of Porto, Porto, Portugal.
[3]Centre for Health Technology and Services Research (CINTESIS), University of Porto, Porto, Portugal.

Monteiro, Hugo;[5] Email: hfmonteiro@arsnorte.min-saude.pt
[5]Research and Planning Department, Regional Health Administration of Northern, Min. of Health Portugal.

Martins, Carlos.[2, 3] Email: carlosmartins20@gmail.com
[2]Department of Community Medicine, Information and Health Decision Sciences (MEDCIDS), Faculty of Medicine, University of Porto, Porto, Portugal.
[3]Centre for Health Technology and Services Research (CINTESIS), University of Porto, Porto, Portugal.

WORD COUNT

3379

Corresponding Author
Susana Silva Pinto
susyapinto@gmail.com
Rua Dr. Plácido da Costa, s/n 4200-450, Porto, Portugal

For peer review only

ABSTRACT

Objectives

To determine the prevalence of AF and to assess how these patients are being cared for: What anticoagulants are being prescribed and are they being prescribed as recommended?

Design

Retrospective longitudinal study.

Setting

This study was conducted in the Regional Health Administration of Northern Portugal.

Participants

This study used a database that included 63526 patients with code K78 of the International Classification of Primary Care between January 2016 and December 2018.

Results

The prevalence of AF among adults over 40 years in the northern region of Portugal was 2.3% in 2016, 2.8% in 2017, and 3% in 2018. From a total of 63526 patients, 95.8% had an indication to receive anticoagulation therapy. Of these, 44326 (72.9%) are being treated with anticoagulants: 17936 (40.5%) were prescribed vitamin K antagonists and 26390 (59.5%) were prescribed non-vitamin K antagonist anticoagulants. On the other hand, 2688 patients of the total (4.2%) had no indication to receive anticoagulation therapy. Of these 2688 patients, 1100 (40.9%) were receiving anticoagulants.

Conclusions

The prevalence of AF is 3%. Here, we report evidence of both undertreatment and overtreatment. Albeit having an indication, a considerable proportion of patients (27.1%) are not anticoagulated, and among AF patients without an indication to receive anticoagulation therapy, a considerable proportion (40.9%) are receiving anticoagulants. The AF-React study brings extremely relevant conclusions to Portugal and follows real-world studies in patients with AF in Europe, presenting some data not yet studied.

Keywords

atrial fibrillation, anticoagulants, prevention, stroke, primary health care.

Strengths and limitations of this study

- The main limitation of this retrospective study is that the AF assessment is based on data coded in the clinical process in Primary Health Care.
- There might be more patients with AF in the northern region of Portugal, but they are not coded at Primary Health Care and, therefore, were not included in this study.
- The lack of ICPC-2 coding for problems such as mechanical prosthesis valve and rheumatic moderate or severe mitral stenosis might be another limitation.
- There might be a small percentage of patients well-medicated with vitamin K antagonists because they are in one of those situations.

  Few of the 63526 patients will be diagnosed with atrial flutter; however, for patients with atrial flutter, anticoagulant therapy is recommended according to the same risk profile used for AF.

For peer review only

- The fact that it is not known when treatment started for patients already diagnosed with AF before 2016 may be a limitation for interpreting the data.

For peer review only

BACKGROUND

Atrial fibrillation (AF) is the most common type of sustained cardiac arrhythmia.(1) The clinical relevance of AF is associated with the loss of effective atrial contractility, resulting in deficient emptying of the left atrial appendage. This process increases the risk of thrombus formation and thromboembolic events.(1)

Due to the ageing of the population, the number of people affected by AF worldwide is projected to exceed 12 million by 2050.(2) In Portugal in 2010, the FAMA study(3) verified an overall prevalence of AF of 2.5% in a population sample older than 40 years, with a significant increase after the age of 70 years.(3)

In Portugal, despite a considerable reduction in ischemic stroke mortality below 70 years (39% reduction) in 2015 compared to 2011, stroke remains the main cause of mortality related to vascular diseases.(4) Patients with AF have a five-fold increased risk of stroke.(5) This arrhythmia is currently responsible for about 15% of stroke cases.(3) Stroke is a major complication associated with AF, which contributes to the morbidity and mortality associated with the disease.(6)

Previous studies have reported that oral anticoagulation with vitamin K antagonists (VKA), such as warfarin, reduces stroke and mortality in AF patients.(7) The non-vitamin K antagonist oral anticoagulants (NOAC) – dabigatran, rivaroxaban, apixaban, edoxaban – have been shown to be superior to warfarin in the prevention of thromboembolic events in patients with non-valvular AF, providing increased safety and an overall reduction in the number of cases of bleeding events(8–11). The cost and morbidity associated with stroke and intracranial haemorrhage are high, so the lower prevalence of these events with NOACs, compared to warfarin, is one factor that might help this drug to become cost-effective relative to warfarin.(12) Furthermore, according to the Portuguese Directorate-General of Health, the reduction in ischemic stroke mortality below 70 years was due to the introduction of NOAC into clinical practice as an antithrombotic therapy for AF. (4)

In a cross-sectional study of patients with AF carried out in eight Family Health Units of Northern Portugal in 2015 (the FATA study),(13) only 56.8% of patients with AF were prescribed adequate oral anticoagulation according to the recommendations of the European Society of Cardiology (ESC) Guidelines in 2010. In the SAFIRA study (2018),(14) 56.3% of patients with AF were not anticoagulated.

There are no randomised controlled trials (RCT) directly comparing NOACs to each other (or to placebo). All RCTs compared a NOAC to warfarin. For example, the RE-LY,(8) ROCKET-AF,(9) ARISTOLETE,(10) and ENGAGE AF-TIMI 48(11) studies included 18113, 14264, 18201, and 21105 patients with AF, respectively. In fact, these trials were crucial for the efficacy and safety evaluation of these drugs and their approval. However, real-world studies might contribute additional relevant evidence because they include epidemiological data and drug prescribing patterns for a larger number of patients in the context of daily clinical practice. In Portugal, there are no real-world studies with a large database of oral anticoagulation in patients with AF.

In Europe, there are some studies with a large database. A study involving databases from 6 European countries concluded that, overall, apixaban and rivaroxaban increased their use during the study period while dabigatran decreased and there was variability in patient characteristics such as comorbidities, potentially interacting drugs, and dose adjustment.(15) Komen et al. (2017) investigated the influence of patient characteristics such as age, stroke, and bleeding risks on decisions for antithrombotic treatment in patients with AF and concluded that apixaban was favoured for elderly and high-risk patients, whereas dabigatran was used in lower-risk patients.(16) Mueller et al. (2019) compared the clinical effectiveness and safety of NOACs in patients with AF in routine clinical practice. All NOACs were similarly effective at preventing strokes and systemic embolisms, while patients being treated with rivaroxaban exhibited the highest bleeding risks.(17)

For peer review only

The AF-React study aims to determine the prevalence of AF in the northern region of Portugal and to assess how patients with AF are being cared for: What anticoagulants are being prescribed and are they being prescribed as recommended?

For peer review only

METHODS

The Portuguese National Health Service is a universal health coverage system and is administratively divided into five regions: North, Centre, Lisbon and Tagus Valley, Alentejo, and Algarve. The entirety of the mainland Portuguese population is enrolled in one of these administrative health regions. Each regional health administration is responsible for providing primary and secondary healthcare to the population living in its geographic area. This retrospective longitudinal study was conducted using data from the Regional Health Administration database of Northern Portugal.

The Department of Studies and Planning of the Regional Health Administration of Northern Portugal built a database containing the following data for the years 2016, 2017, and 2018: population enrolled in the Regional Health Administration of Northern Portugal, number of patients diagnosed with other ICPC-2 coded health problems,(18) and last record available of body mass index, cardiovascular risk score, and glomerular filtration rate. In fact, this is one-off data (once a year) for all variables (age, gender, professional situation, ICPC-2 coded health problems, body mass index, cardiovascular risk score, and glomerular filtration rate) except the anticoagulants prescription history, for which we had longitudinal information with the date of each prescription.

To identify AF patients, the International Classification of Primary Care (ICPC-2) code for AF, K78, was used.(18) Therefore, we included all adults (age $\geq$ 18 years) with the code K78 between January and December of the years 2016, 2017, and 2018. For each year, either previous AF diagnosed patients or new AF diagnosed patients were included, so the database comprises both incident and prevalent patients.

The prevalence of AF was calculated for the population enrolled in the Regional Health Administration of Northern Portugal in 2016, 2017, and 2018.

The CV risk (cardiovascular risk) was defined in four stages based on SCORE (Systematic Coronary Risk Evaluation), as low, moderate, high, and very high, if less than 1%, between 1% and 5%, between 5% and 10%, and greater than or equal to 10%, respectively, for patients between 40 and 65 years old.

The stages of CKD were defined based on the glomerular filtration rate (GFR) calculated by the equation of *Cockcroft-Gault*: stage 1, GFR $\geq$ 90 ml/min; stage 2, GFR]90-60] ml/min; stage 3, GFR]60-30] ml/min; stage 4, GFR [30-15] ml/min; and stage 5, GFR < 15ml/min. The high and very high stages of CV risk were based on the codification of health problems by ICPC-2 and the patient's GFR registered in the clinical process.

The body mass index was defined in six categories: <18,5 kg/m^2 – underweight; [18-25[kg/m^2 – normal; [25-30[kg/m^2 – overweight; [30-35[kg/m^2 – obese class I; [35-40[kg/m^2 – obese class II; and $\geq$ 40 kg/m^2 kg/m^2 – obese class III.

The CHA_2DS_2-VASc score was calculated according to the ESC guidelines in 2016 for AF(19), as follows by ICPC-2 coding (18): C-Congestive heart failure (K77): 1 point; H-Hypertension (K86 or K87): 1 point; A_2-Age > 75 years or older: 2 points; D-Diabetes mellitus (T89 or T90): 1 point; S_2-Stroke (K89, K90, or K91): 2 points; V-Vascular disease (K75 or K92): 1 point; A-Age 65-74 years: 1 point; and Sc-Sex category (female): 1 point. According to the 2016 ESC guidelines,(19) we considered a CHA_2DS_2-VASc score $\geq$ 1 point in a male and a CHA_2DS_2-VASc score $\geq$ 2 points in a female to be an indication of anticoagulation.

The categorical variables were described using absolute and relative frequencies, n (%). The normally distributed continuous variables were described by the mean and respective standard deviation, mean±SD, and by the minimum (min) and maximum (máx) values. In the case of continuous variables not normally distributed, the data are presented by the median and respective interquartile range, Med [Q_1; Q_3], where Q_1 is the first quartile and Q_3 is the third quartile. The normality was verified by observing the histograms.

For peer review only - http://bmjopen.bmj.com/site/about/guidelines.xhtml

The database was exported to Microsoft Excel 2016® and the statistical analysis was performed using SPSS Statistics 25.0® and software R. Data were obtained without aggregation at the individual level for general characterisation analysis.

The data processing was carried out through the Department of Studies and Planning of the Regional Health Administration of Northern Portugal (Ministry of Health, Portugal). The data were extracted from the server through an anonymised data processing and editing platform and delivered securely (and in accordance with legal regulations and due approval) to the principal investigator. Patients and the public were not involved in the development of the research question.

For peer review only

PATIENT AND PUBLIC INVOLVEMENT

Patients and the public were not involved in the development of the research question.

RESULTS

A total of 63526 patients were identified as having the diagnosis of atrial fibrillation/flutter (ICPC-2, K78 code) in the northern region of Portugal. The average age was 76.5 ± 10.6 years (range from 18 to 107 years) and 53% were female. Table 1 provides a detailed characterisation of this patient group. Considering the 2077599 adults over 40 years enrolled in 2018 in the Regional Health Administration of Northern Portugal, the prevalence of AF was 3.044% (Table 2). In 2017, among the 1943813 adults over 40 years enrolled in the Regional Health Administration of Northern Portugal, the prevalence of AF was 2.787%. In 2016, among the 2024390 adults over 40 years enrolled in the Regional Health Administration of Northern Portugal, the prevalence of AF was 2.295%.

From a total of 63526 patients, 60838 (95.8%) had an indication to receive anticoagulation therapy: male with CHA_2DS_2-VASc score $\geq$ 1 and female with CHA_2DS_2-VASc score $\geq$ 2. Among these 60838 patients, 16512 (27.1%) were not being treated with anticoagulants, and 79% of those 16512 had a CHA_2DS_2-VASc score of 3 or higher.

Among the 44326 being treated with anticoagulants, 17936 (40.5%) were prescribed VKAs (warfarin and acenocoumarol) and 26390 (59.5%) with NOACs (dabigatran, rivaroxaban, apixaban, and edoxaban). The most commonly prescribed drug in medicated patients was warfarin (31%), followed by rivaroxaban (23.6%), apixaban (20.3%), dabigatran (12.6%), acenocoumarol (9.6%), and edoxaban (2.9%).

The CHA_2DS_2-VASc score has the following distribution: zero points (2.6%), 1–3 points (40.7%), 4–5 points (43.3%), and $\geq$ 6 points (13.4%). From the total of 30089 males, 1658 (5.5%) had a CHA_2DS_2-VASc score of zero, and from a total of 33437 females, 1030 (3.1%) had a CHA_2DS_2-VASc score of 1. This means that these 2688 (4.2%) patients of the total had no indication to receive anticoagulation therapy. Of these 2688 patients, 1100 were receiving anticoagulants (Table 3).

The distributions of the different drugs by CHA_2DS_2-VASc score and gender are shown in Table 4. For males, rivaroxaban (15.0%) and apixaban (8.1%) were more often prescribed than warfarin (7.7%) in the group with a CHA_2DS_2-VASc score of zero. In particular, apixaban was more prescribed than rivaroxaban in groups with CHA_2DS_2-VASc scores of 5 or 6 (15.2% *vs* 14.7% and 13.4% *vs* 13.2%, respectively). For females, apixaban was more prescribed than rivaroxaban in the groups with CHA_2DS_2-VASc scores of 7, 8, or 9 (17.5% *vs* 19.7%, 16.7% *vs* 13.0%, and 13.9% *vs* 13.1%, respectively).

The CV risk was available for 6531 (72.7%) of the patients between 40 and 65 years old. The CV risk values, for patients in that age group, were distributed as follows: low – 1.0%, moderate – 40.3%, high – 3.0%, and very high – 46.8%.

The glomerular filtration rate was available for 52207 patients (82.2%) and followed this distribution: stage 1 (21.2%); stage 2 (37.7%); stage 3 (37.0%); stage 4 (4.0%); and stage 5 (0.1%). The VKAs (warfarin and acenocoumarol) were more prescribed than NOACs (dabigatran, rivaroxaban, apixaban, and edoxaban) in stages 4 and 5. The distribution of patients for CKD stages by respective drugs is represented in Table 5.

Of 63526 patients with AF, between 2016 and 2018, 17936 (28.2%) were always prescribed VKA and 21854 (34.4%) were always prescribed NOAC. In particular, 4133 patients (6.5% of the total) had a single recorded prescription for any anticoagulant: warfarin – 1496 (36.2%), rivaroxaban – 882 (21.34%), apixaban – 815 (19.71%), acenocoumarol – 397 (9.61%), dabigatran – 370 (8.95%), and edoxaban – 173 (4.19%). Furthermore, 3508 (5.5%) were prescribed VKA and switched to NOAC, 1504 (2.4%) were prescribed NOAC and switched to another NOAC, and 305 (0.5%) were prescribed NOAC and switched to VKA. The following patients were always prescribed the same NOAC: rivaroxaban – 8801 (22.18%), apixaban – 7052 (17.78%), dabigatran – 5219 (13.16%), and edoxaban – 782 (1.97%).

For peer review only

245 The prevalence of each of the comorbidities studied in patients with AF is represented in Table 6. We found that the most prevalent comorbidities associated with AF were hypertension (77.2%), dyslipidemia (52.1%), diabetes (28.5%), and heart failure (27.6%).

For peer review only

DISCUSSION

Summary

In the AF-React study, the prevalence of AF was 3% in 2018 in population aged 40 years or older. A considerable proportion of patients, with an indication for anticoagulation therapy, are not anticoagulated (27.1%), and among AF patients without indication to receive anticoagulation therapy, a considerable proportion (40.9%) are receiving anticoagulants.

Comparison with existing literature

The prevalence of AF found in this study is similar to that of the FAMA study(3) (3% *vs* 2.5%), and higher than that of the FATA study(13) (3% *vs* 1.3%). The FAMA study(3) used a representative sample of the Portuguese population over 40 years old to study the prevalence of AF in 2010. The AF disease was determined using a 12-lead electrocardiogram. The FATA study(13) is an observational cross-sectional study from 2014 that included all patients aged 30 or above diagnosed with AF, enrolled in one of the eight Family Health Units of a northern region of Portugal. The higher values found in our study, as compared to the FATA study,(13) corroborate our results: The prevalence of AF is increasing with time in the northern region of Portugal. As far as following expectations, this might also be related to the improvement of the coding process of the problems in the clinical process. It was found that the majority of AF patients in this study are elderly, as in the ATRIA study(4) (44.8% were aged 80 years or older *vs* 45% were aged 75 years or older).

The CHA_2DS_2-VASc scores found in our study are similar to those of the FATA study(13): zero points (2.6% *vs* 2.3%), 1–3 points (40.7% *vs* 40.9%), 4–5 points (43.3% *vs* 41.6%), and ≥ 6 (13.4% *vs* 14.3%). In our study, 44326 (72.9%) patients were adequately anticoagulated, which is an improvement relative to the FAMA,(3) FATA,(13) SAFIRA,(14) and REACH (20) studies (38%, 56.8%, 43.7%, and 54%, respectively). These studies were published prior to the 2016 ESC guidelines.(19) The guidelines advise that NOAC should be considered for men with a CHA_2DS_2-VASc score ≥ 1 and women with a score ≥ 2, which might have contributed to the improvement in anticoagulation in AF patients. It should be noted that, in our study, 79% of patients with AF who are not anticoagulated have a high thrombotic risk (CHA_2DS_2-VASc score ≥ 3). This information is of most importance because clinicians should be proactive in the prevention of stroke in patients with AF.

In addition to evidence of anticoagulation undertreatment in AF patients, overtreatment is also found. In our study, 40.9% of AF patients who do not have an indication for anticoagulation are taking anticoagulants. Similar overtreatment rates occur in the REACH study,(20) where 43% of patients with AF and not indicated for anticoagulation were receiving anticoagulants. The FAMA,(3) FATA,(13) and SAFIRA(14) studies did not report overtreatment rates. A possible explanation for this overtreatment rate in our study might be a misuse of the CHA_2DS_2-VASc score. Some of these patients might also be prescribed an anticoagulation agent for other clinical reasons. Hess, P.L. et al. (2014) identified a broad range of barriers to oral anticoagulation, including knowledge gaps about stroke risk and the relative risks and benefits of anticoagulant therapies; lack of awareness regarding the potential use of NOAC agents for VKA-unsuitable patients; lack of recognition of expanded eligibility for oral anticoagulation; lack of availability of reversal agents and the difficulty of anticoagulant effect monitoring for the NOACs; concerns with the bleeding risk of anticoagulant therapy, especially with the NOACs and particularly in the setting of dual antiplatelet therapy; suboptimal time in therapeutic range for VKA; and costs and insurance coverage.(21)

Although there has been a positive evolution regarding the number of patients with anticoagulated AF, warfarin remains the most prescribed anticoagulant. As noted before, this is not concordant with the 2016 ESC guidelines.(19) When analysing the distribution of drugs by CHA_2DS_2-VASc score and gender, it should be noted that in men who are not indicated to be anticoagulated (CHA_2DS_2-VASc score of zero), the most prescribed drug is rivaroxaban, which might result from another

For peer review only

therapeutic indication, such as deep venous thrombosis. On the other hand, acenocoumarol is more prescribed than dabigatran both in men with CHA_2DS_2-VASc scores of 7 and 8 and in women with a CHA_2DS_2-VASc score of 8. These results might be explained by the fact that clinicians prefer drugs with which they have more experience, such as VKA in patients with a very high thrombotic risk. However, this does not follow the most current guidelines.

The SAFIRA(14) study showed a very high prevalence of CV risk factors in patients with AF. In Portugal, SCORE is recommended for use in calculating CV risk in those between 40 and 65 years old.(22,23) However, we found no study that calculated CV risk by SCORE. The CV risk of patients aged 40–65 years was considered, with most patients having a very high CV risk. In fact, according to the 2016 ESC guidelines,(19) most patients with AF have many other comorbidities, so we consider this to be a reason for having a much larger number of very high risk than high risk patients in our study.

The SAFIRA(14) study found that 24.7% of the patients were incorrectly medicated (inadequate dose, inadequate number of doses, or creatinine clearance incompatible with the use of NOAC), but it did not discriminate patients by stages of CKD. The AF-React study showed that NOACs are being prescribed in stage 5 of CKD, which was contraindicated by the guidelines in force at the date of prescription (between 2016 to 2018).(18,24) In stage 4 of CKD, the 2016 ESC Guidelines(19) do not recommend the use of NOACs; the EHRA practical guide(24) recommends dose adjustment for apixaban, rivaroxaban, and edoxaban and does not recommend the use of dabigatran. This data opens the door to a more detailed analysis in the future about the prescriptions in stage 4.

Limitations

The main limitation of this retrospective study is that the AF assessment is based on data coded in the clinical process in Primary Health Care. There might be more patients with AF in the northern region of Portugal, but they are not coded at Primary Health Care and, therefore, were not included in this study. Hence, strict coding by all family doctors is important. Another limitation might be the lack of ICPC-2 coding for problems such as mechanical prosthesis valve and rheumatic moderate or severe mitral stenosis(19). There might be a small percentage of patients who are well medicated with vitamin K antagonists because they are in one of those situations.

The code K78, ICPC-2 included the diagnosis of AF and atrial flutter. Few of the 63526 patients will be diagnosed with atrial flutter, so this is a coding limitation of ICPC-2. However, this limitation does not invalidate the results of our study because, for patients with atrial flutter, anticoagulant therapy is recommended according to the same risk profile used for AF.(25)

The fact that it is not known when treatment started for patients already diagnosed with AF before 2016 may be a limitation for interpreting the data. Besides that, the database didn’t allow a detailed timing and sequencing of diagnosis, which might have implications for interpreting our findings.

Implications for research and practice

The AF-React study made it possible to determine the prevalence of AF and to evaluate how patients with AF are treated in northern Portugal. Thus, the AF-React study brings extremely relevant conclusions to Portugal and follows real-world studies in patients with AF in Europe, presenting some data not yet studied: distribution of patients for CHA_2DS_2-VASc score value, gender and respective drugs, classification of CV risk and distribution of patients for chronic kidney disease stages by respective drugs. For the future, it would be necessary for health authorities to allow continued access to these data sets, including mortality data, to draw conclusions (based on the real world) about the impact of the new treatments on patient-oriented outcomes.

For peer review only

FUNDING

This article was supported by National Funds through FCT - Fundação para a Ciência e a Tecnologia I.P., within CINTESIS, R&D Unit (reference UIDB/IC/4255/2020). The authors have received financial support of €4,400 EUR from the #H4A Primary Healthcare Research Network Scholars Program research support.

For peer review only

ETHICAL APPROVAL

The study protocol was approved by the Health Ethics Committee of the Regional Health Administration of Northern Portugal.

For peer review only

COMPETING INTERESTS

The authors declared no potential conflicts of interest with respect to the research, authorship, and/or publication of this article.

For peer review only

ACKNOWLEDGEMENTS

A special thanks to all primary healthcare professionals who in their daily work in the Portuguese Health System fulfil the clinical records and allow researchers like us to obtain evidence that will help us to improve the health of the Portuguese.

For peer review only

AUTHOR CONTRIBUTIONS

SSP and CM contributed to the conception of the work; all authors contributed to the design of the study. HM contributed to the data acquisition process; SPP, AT, and TH contributed to the analysis and interpretation of the data. SSP drafted the manuscript, HM, AT, TH, and CM critically revised the manuscript. All authors gave final approval and agreed to be accountable for all aspects of the work, ensuring integrity and accuracy.

For peer review only

DATA SHARING STATEMENT

Data are available upon reasonable request.

For peer review only

REFERENCES

1. Falk RH. Atrial fibrillation. *N Engl J Med* 2001;344(14):1067–78.

2. Shafeeq H, Tran TH. New oral anticoagulants for atrial fibrillation: are they worth the risk? *P T Peer-Rev J Formul Manag* 2014;39(1):54–64.

3. Bonhorst D, Mendes M, Adragão P, et al. Prevalence of atrial fibrillation in the Portuguese population aged 40 and over: the FAMA study. *Rev Port Cardiol.* 2010;29(3):331–50.

4. Ferreira RC, Macedo ME, Pinto F, et al. PROGRAMA NACIONAL PARA AS DOENÇAS CÉREBRO-CARDIOVASCULARES 2017 [Internet]. Direção Geral da Saúde; 2017.

5. da Silva RMFL. Novel oral anticoagulants in non-valvular atrial fibrillation. *Cardiovasc Hematol Agents Med Chem* 2014;12(1):3–8.

6. Alonso A, Norby FL. Predicting Atrial Fibrillation and Its Complications. *Circ J Off J Jpn Circ Soc* 2016;80(5):1061–6.

7. Aguilar MI, Hart R. Oral anticoagulants for preventing stroke in patients with non-valvular atrial fibrillation and no previous history of stroke or transient ischemic attacks. Cochrane Database Syst Rev. 2005;(3):CD001927.

8. Connolly SJ, Ezekowitz MD, Yusuf S, et al. Dabigatran versus warfarin in patients with atrial fibrillation. *N Engl J Med* 2009;361(12):1139–51.

9. Patel MR, Mahaffey KW, Garg J, et al. Rivaroxaban versus warfarin in nonvalvular atrial fibrillation. *N Engl J Med* 2011;365(10):883–91.

10. Granger CB, Alexander JH, McMurray JJV, et al. Apixaban versus warfarin in patients with atrial fibrillation. *N Engl J Med* 2011;365(11):981–92.

11. Giugliano RP, Ruff CT, Braunwald E, et al. Edoxaban versus warfarin in patients with atrial fibrillation. *N Engl J Med* 2013;369(22):2093–104.

12. Matchar DB, Samsa GP, Cohen SJ, et al. Improving the quality of anticoagulation of patients with atrial fibrillation in managed care organizations: results of the managing anticoagulation services trial. *Am J Med* 2002;113(1):42–51.

13. Gomes E, Campos R, Morais R, et al. [FATA Study: prevalence of atrial fibrillation and antithrombotic therapy in primary health care in a northern city of Portugal]. *Acta Med Port* 2015;28(1):35–43.

14. Monteiro P. Estudo Safira: reflexões sobre a prevalência e os padrões de tratamento de fibrilhação auricular e risco cardiovascular em 7500 indivíduos com 65 ou mais anos. *Rev Port Cardiol* 2018;37(4):307–13.

15. Ibáñez L, Sabaté M, Vidal X, et al. Incidence of direct oral anticoagulant use in patients with nonvalvular atrial fibrillation and characteristics of users in 6 European countries (2008–2015): A cross-national drug utilization study. *Br J Clin Pharmacol* 2019;85(11):2524–39.

For peer review only

16. Komen J, Forslund T, Hjemdahl P, et al. Factors associated with antithrombotic treatment decisions for stroke prevention in atrial fibrillation in the Stockholm region after the introduction of NOACs. *Eur J Clin Pharmacol* 2017;73(10):1315–22.

17. Mueller T, Alvarez-Madrazo S, Robertson C, et al. Comparative safety and effectiveness of direct oral anticoagulants in patients with atrial fibrillation in clinical practice in Scotland. *Br J Clin Pharmacol* 2019;85(2):422–31.

18. World Organization of National Colleges, Academies, and Academic Associations of General Practitioners/Family Physicians, editor. ICPC-2: international classification of primary care. 2nd ed. Oxford ; New York: Oxford University Press; 1998. 190 p. (Oxford medical publications).

19. Kirchhof P, Benussi S, Kotecha D, et al. 2016 ESC Guidelines for the management of atrial fibrillation developed in collaboration with EACTS. *Eur Heart J* 2016;37(38):2893–962.

20. Ruff CT, Bhatt DL, Steg PG, et al. Long-term cardiovascular outcomes in patients with atrial fibrillation and atherothrombosis in the REACH Registry. *Int J Cardiol* 2014;170(3):413–8.

21. Hess PL, Mirro MJ, Diener H-C, et al. Addressing barriers to optimal oral anticoagulation use and persistence among patients with atrial fibrillation: Proceedings, Washington, DC, December 3-4, 2012. *Am Heart J* 2014;168(3):239-247.e1.

22. European Association for Cardiovascular Prevention & Rehabilitation, Reiner Z, Catapano AL, De Backer G, et al. ESC/EAS Guidelines for the management of dyslipidaemias: the Task Force for the management of dyslipidaemias of the European Society of Cardiology (ESC) and the European Atherosclerosis Society (EAS). *Eur Heart J* 2011;32(14):1769–818.

23. Authors/Task Force Members: Piepoli MF, Hoes AW, Agewall S, et al. 2016 European Guidelines on cardiovascular disease prevention in clinical practice: The Sixth Joint Task Force of the European Society of Cardiology and Other Societies on Cardiovascular Disease Prevention in Clinical Practice (constituted by representatives of 10 societies and by invited experts) Developed with the special contribution of the European Association for Cardiovascular Prevention & Rehabilitation (EACPR). *Atherosclerosis* 2016;252:207–74.

24. Steffel J, Verhamme P, Potpara TS, et al. The 2018 European Heart Rhythm Association Practical Guide on the use of non-vitamin K antagonist oral anticoagulants in patients with atrial fibrillation. *Eur Heart J* 2018;39(16):1330–93.

25. January CT, Wann LS, Calkins H, et al. 2019 AHA/ACC/HRS Focused Update of the 2014 AHA/ACC/HRS Guideline for the Management of Patients With Atrial Fibrillation: A Report of the American College of Cardiology/American Heart Association Task Force on Clinical Practice Guidelines and the Heart Rhythm Society in Collaboration With the Society of Thoracic Surgeons. *Circulation* [Internet]. 2019.

For peer review only

449 **TABLES**

450 **Table 1. Characterization of the atrial fibrillation patients diagnosed in the Regional Health**
451 **Administration of Northern Portugal (n= 63526).**

**Gender,** *n (%)*	
Female	33437 (52.6%)
Male	30089 (47.4%)
**Age (years),** *mean±SD, min, máx*	76.5 ± 10.6, 18, 107
**Age groups,** *n(%)*	
<40 years	286 (0.5)
40-49 years	812 (1.3)
50-59 years	3286 (5.2)
60-69 years	10089 (15.9)
70-79 years	20602 (32.4)
≥ 80 years	28451 (44.8)
**Professional situation,** *n(%)*	
Active	13553 (21.3)
Not Active	4957 (7.8)
Student	54 (0.1)
Retired	44685 (70.3)
Unknown	277 (0.5)
**Body Mass Index,** *n(%)*	9448 (14.9%)
**Body Mass Index[#],** *Med [Q_1; Q_3]*	29.1 [26.2; 32.6]
**Body Mass Index Categories,** *n(%)*	
**Underweight**	29 (0.3)
**Normal**	1272 (13.5)
**Overweight**	4126 (43.7)
**Obese Class I**	2725 (28.8)
**Obese Class II**	1297 (13.7)
**Glomerular Filtration Rate*,** *n(%)*	
**Value available**	52207 (82.2%)
**Missing record**	11319 (17.8%)
**Stage of Chronic Kidney Disease**	
**1**	11044 (21.2%)
**2**	19693 (37.7%)
**3**	19307 (37.0%)
**4**	2110 (4.0%)
**5**	53 (0.1%)
**Cardiovascular risk for patients with age between 40-65 years (n =8985),** *n(%)*	
**Value available**	6531 (72.7%)
**Missing record**	2454 (27.3%)

For peer review only

**Classification of Cardiovascular Risk[¥] (n =6531),** *n(%)*	
**Low**	650 (10.0%)
**Moderate**	2631 (40.3%)
**High**	194 (3.0%)
**Very high**	3056 (46.7%)
**CHA_2DS_2-VASc score,** *n(%)*	
**0**	1658 (2.6%)
**1**	3967 (6.2%)
**2**	8065 (12.7%)
**3**	13811 (21.7%)
**4**	16152 (25.4%)
**5**	11388 (17.9%)
**6**	5520 (8.7%)
**7**	2196 (3.5%)
**8**	685 (1.1%)
**9**	84 (0.1%)

[#] The body mass index is defined under six categories: <18,5 kg/m2 – underweight; [18-25[kg/m2 – normal; [25-30[kg/m2 – overweight; [30-35[kg/m2 – obese class I; [35-40[kg/m2 – obese class II; ≥ 40 kg/m2 kg/m2 – obese class III.

* The stages of CKD were defined based on the glomerular filtration rate (GFR) calculated by the equation of *Cockcroft-Gault*: stage 1, GFR ≥ 90 ml/min; stage 2, GFR]90-60] ml/min; stage 3, GFR]60-30] ml/min, stage 4, GFR [30-15] ml/min; and stage 5, GFR < 15ml/min.

¥ The cardiovascular risk was defined under four stages that were based on SCORE (Systematic Coronary Risk Evaluation), as low, moderate, high, and very high, if less than 1%, between 1% and 5%, between 5% and 10%, and greater than or equal to 10%.

For peer review only

Table 2. Prevalence (%) of atrial fibrillation in the northern region of Portugal between 2016 and 2018.

		< 40 years	40-49 years	50-59 years	60-69 years	70-79 years	≥ 80 years	Over 40 years
**2016**	AF patients (n)	200	546	2184	7061	15089	21576	46456
	Population[#] (n)	1064388	593200	502021	438432	286658	204079	2024390
	Prevalence (%)	**0.019**	**0.092**	**0.435**	**1.611**	**5.264**	**10.572**	**2.295**
**2017**	AF patients (n)	239	678	2647	8442	17665	24746	54178
	Population[$] (n)	952914	506129	527554	433722	258239	218169	1943813
	Prevalence (%)	**0.025**	**0.134**	**0.502**	**1.946**	**6.841**	**11.343**	**2.787**
**2018**	AF patients (n)	286	812	3286	10089	20602	28451	63240
	Population[£] (n)	957693	588852	530424	432439	307823	218061	2077599
	Prevalence (%)	**0.030**	**0.138**	**0.620**	**2.333**	**6.693**	**13.047**	**3.044**

[#]Population enrolled in primary health care in northern Portugal based on the age pyramid of Regional Health Administration of Northern Portugal in 2016. [$]Population enrolled in primary health care in northern Portugal based on the age pyramid of Regional Health Administration of Northern Portugal in 2017. [£]Population enrolled in primary health care in northern Portugal based on the age pyramid of Regional Health Administration of Northern Portugal in 2018.

For peer review only

Table 3. Medicated and non-medicated patients with and without a recommendation for anticoagulation.

Recommendation[#]	Medicated, n (%)		Total, n
	Yes	No	
**Yes**	44326 (72.9)	16512 (27.1)	60838
**No**	1100 (40.9)	1588 (59.1)	2688
**Total**	45426 (71.5)	18100 (28.5)	63526

[#]Oral anticoagulation was considered in a male with a CHA_2DS_2-VASc score ≥ 1 point and in a female with a score ≥ 2 points, according to 2016 European Society of Cardiology Guidelines for the management of atrial fibrillation developed in collaboration with European Association of Cardio-Thoracic Surgery.

For peer review only

Table 4. Distribution of patients for CHA_2DS_2-VASc score value, gender, and respective drugs.

CHA_2DS_2-VASc score	Drugs, *n (%)*								
	No OAC*	NOAC[#] group					VKA group		
	Total	Total	rivaroxaban	apixaban	dabigatran	edoxaban	Total	warfarin	acenocoumarol
Male									
**0**	990 (59.7)	500 (30.2)	248 (15.0)	134 (8.1)	88 (5.3)	30 (1.8)	168 (10.1)	127 (7.7)	41 (2.5)
**1**	1069 (36.4)	1313 (44.7)	623 (21.2)	344 (11.7)	276 (9.4)	70 (2.4)	555 (18.9)	426 (14.5)	129 (4.4)
**2**	1646 (28.6)	2648 (46.0)	1125 (19.5)	801 (13.9)	590 (10.2)	132 (2.3)	1468 (25.5)	1099 (19.1)	369 (6.4)
**3**	2132 (25.3)	3742 (44.5)	1531 (18.2)	1210 (14.4)	823 (9.8)	178 (2.1)	2537 (30.2)	1955 (23.2)	582 (6.9)
**4**	1549 (24.8)	2572 (41.2)	1037 (16.6)	877 (14.0)	547 (8.8)	111 (1.8)	2127 (34.0)	1636 (26.2)	491 (7.9)
**5**	798 (25.0)	1301 (40.7)	471 (14.7)	486 (15.2)	286 (9.0)	58 (1.8)	1095 (34.3)	813 (25.5)	282 (8.8)
**6**	377 (27.1)	502 (36.1)	183 (13.2)	187 (13.4)	102 (7.3)	30 (2.2)	512 (36.8)	371 (26.7)	141 (10.1)
**7**	96 (23.1)	164 (39.4)	61 (14.7)	58 (13.9)	38 (9.1)	7 (1.7)	156 (37.5)	116 (27.9)	40 (9.6)
**8**	19 (26.4)	25 (34.7)	12 (16.7)	8 (11.1)	2 (2.8)	3 (4.2)	28 (38.9)	20 (27.8)	8 (11.1)
**Subtotal**	8676 (28.8)	12767 (42.4)	5291 (17.6)	4105 (13.6)	2752 (9.1)	619 (2.1)	8646 (28.7)	6563 (21.8)	2083 (6.9)
Female									
**1**	598 (58.1)	272 (26.4)	118 (11.5)	88 (8.5)	49 (4.8)	17 (1.7)	160 (15.5)	125 (12.1)	35 (3.4)
**2**	757 (32.9)	1004 (43.6)	418 (18.2)	345 (15.0)	189 (8.2)	52 (2.3)	542 (23.5)	432 (18.8)	110 (4.8)
**3**	1544 (28.6)	2399 (44.4)	951 (17.6)	781 (14.5)	515 (9.5)	152 (2.8)	1457 (27.0)	1130 (20.9)	327 (6.1)
**4**	2555 (25.8)	4460 (45.0)	1708 (17.2)	1565 (15.8)	979 (9.9)	208 (2.1)	2889 (29.2)	2195 (22.2)	694 (7.0)
**5**	2142 (26.1)	3369 (41.3)	1287 (15.7)	1281 (15.6)	694 (8.5)	152 (1.9)	2638 (32.4)	2035 (24.8)	603 (7.4)
**6**	1117 (27.1)	1666 (40.3)	627 (15.2)	623 (15.1)	330 (8.0)	86 (2.1)	1346 (32.6)	1024 (24.8)	322 (7.8)
**7**	525 (29.5)	722 (40.6)	232 (13.0)	312 (17.5)	150 (8.4)	28 (1.6)	533 (29.9)	398 (22.4)	135 (7.6)
**8**	161 (26.3)	257 (41.9)	85 (13.9)	121 (19.7)	43 (7.0)	8 (1.3)	195 (31.8)	148 (24.1)	47 (7.7)
**9**	25 (29.8)	33 (39.3)	11 (13.1)	14 (16.7)	6 (7.1)	2 (2.4)	26 (31.0)	23 (27.4)	3 (3.6)
**Subtotal**	9424 (28.2)	14227 (42.5)	5437 (16.3)	5130 (15.3)	2955 (8.8)	705 (2.1)	9786 (29.3)	7510 (22.5)	2276 (6.8)
**Total**	18100 (28.5)	26994 (42.5)	10728 (16.9)	9235 (14.5)	5707 (9.0)	1324 (2.1)	18432 (29.0)	14073 (22.2)	4359 (6.9)

*OAC: oral anticoagulation
[#] NOAC: non-vitamin K antagonist oral anticoagulants
[$] VKA: vitamin K antagonists

For peer review only

Table 5. Distribution of patients for Chronic Kidney Disease' (CKD) stages by respective drugs

Stages CKD	Drugs, *n (%)*					
	**rivaroxaban**	**apixaban**	**dabigatran**	**edoxaban**	**warfarin**	**acenocoumarol**
**1**	2164 (27.5)	1531 (19.4)	1081 (13.7)	279 (3.5)	2133 (27.1)	691 (8.8)
**2**	3673 (24.7)	2944 (19.8	1980 (13.3)	434 (2.9)	4428 (29.8)	1386 (9.3)
**3**	3184 (22.3)	3139 (22.0)	1649 (11.6)	394 (2.8)	4504 (31.6)	1393 (9.8)
**4**	202 (14.8)	338 (24.7)	86 (6.3)	28 (2.0)	557 (40.7)	157 (11.5)
**5**	4 (11.1)	11 (30.6)	1 (2.8)	1 (2.8)	17 (47.2)	2 (5.6)
**Total**	9227 (24)	7963 (20.7)	4797 (12.5)	1136 (3.0)	11639 (30.3)	3629 (9.5)

For peer review only

Table 6. Comorbidities by the International Classification of Primary Care – 2nd Edition.

Comorbidities	n (%)
chronic alcohol abuse (P15)	2392 (3.8)
tobacco abuse (P17)	2880 (4.5)
lipid disorder (T93)	33095 (52.1)
atherosclerosis/PVD (K92)	4617 (7.3)
diabetes insulin dependente (T89 + T90)	1551 (2.4)
diabetes non-insulin dependente (T90)	16553 (26.1)
coronary heart disease ischaemic heart disease w. angina (K74)	3370 (5.3)
ischaemic heart disease w/o angina (K76)	3185 (5.0)
acute myocardial infarction (K75)	2450 (3.9)
hypertension complicated (K87)	17598 (27.7)
hypertension uncomplicated (K86)	31466 (49.5)
heart failure (K77)	17530 (27.6)

For peer review only

STROBE Statement—Checklist of items that should be included in reports of ***cohort studies***

	Item No	Recommendation	Page No
**Title and abstract**	1	(*a*) Indicate the study's design with a commonly used term in the title or the abstract	2
		(*b*) Provide in the abstract an informative and balanced summary of what was done and what was found	2
**Introduction**			
Background/rationale	2	Explain the scientific background and rationale for the investigation being reported	4
Objectives	3	State specific objectives, including any prespecified hypotheses	4
**Methods**			
Study design	4	Present key elements of study design early in the paper	5
Setting	5	Describe the setting, locations, and relevant dates, including periods of recruitment, exposure, follow-up, and data collection	5
Participants	6	(*a*) Give the eligibility criteria, and the sources and methods of selection of participants. Describe methods of follow-up	5
		(*b*) For matched studies, give matching criteria and number of exposed and unexposed	
Variables	7	Clearly define all outcomes, exposures, predictors, potential confounders, and effect modifiers. Give diagnostic criteria, if applicable	5
Data sources/ measurement	8*	For each variable of interest, give sources of data and details of methods of assessment (measurement). Describe comparability of assessment methods if there is more than one group	5
Bias	9	Describe any efforts to address potential sources of bias	NA
Study size	10	Explain how the study size was arrived at	5
Quantitative variables	11	Explain how quantitative variables were handled in the analyses. If applicable, describe which groupings were chosen and why	5
Statistical methods	12	(*a*) Describe all statistical methods, including those used to control for confounding	5
		(*b*) Describe any methods used to examine subgroups and interactions	
		(*c*) Explain how missing data were addressed	
		(*d*) If applicable, explain how loss to follow-up was addressed	
		(*e*) Describe any sensitivity analyses	
**Results**			
Participants	13*	(a) Report numbers of individuals at each stage of study—eg numbers potentially eligible, examined for eligibility, confirmed eligible, included in the study, completing follow-up, and analysed	6
		(b) Give reasons for non-participation at each stage	
		(c) Consider use of a flow diagram	
Descriptive data	14*	(a) Give characteristics of study participants (eg demographic, clinical, social) and information on exposures and potential confounders	6, 17
		(b) Indicate number of participants with missing data for each variable of interest	
		(c) Summarise follow-up time (eg, average and total amount)	
Outcome data	15*	Report numbers of outcome events or summary measures over time	6, 17

For peer review only - http://bmjopen.bmj.com/site/about/guidelines.xhtml

Main results	16	(*a*) Give unadjusted estimates and, if applicable, confounder-adjusted estimates and their precision (eg, 95% confidence interval). Make clear which confounders were adjusted for and why they were included<br>(*b*) Report category boundaries when continuous variables were categorized<br>(*c*) If relevant, consider translating estimates of relative risk into absolute risk for a meaningful time period	6, 20, 21
Other analyses	17	Report other analyses done—eg analyses of subgroups and interactions, and sensitivity analyses	6, 18, 19, 20
**Discussion**			
Key results	18	Summarise key results with reference to study objectives	7
Limitations	19	Discuss limitations of the study, taking into account sources of potential bias or imprecision. Discuss both direction and magnitude of any potential bias	3
Interpretation	20	Give a cautious overall interpretation of results considering objectives, limitations, multiplicity of analyses, results from similar studies, and other relevant evidence	7-8
Generalisability	21	Discuss the generalisability (external validity) of the study results	7-8
**Other information**			
Funding	22	Give the source of funding and the role of the funders for the present study and, if applicable, for the original study on which the present article is based	9

*Give information separately for exposed and unexposed groups.

Note: An Explanation and Elaboration article discusses each checklist item and gives methodological background and published examples of transparent reporting. The STROBE checklist is best used in conjunction with this article (freely available on the Web sites of PLoS Medicine at http://www.plosmedicine.org/, Annals of Internal Medicine at http://www.annals.org/, and Epidemiology at http://www.epidem.com/). Information on the STROBE Initiative is available at http://www.strobe-statement.org.

For peer review only
